# DREAMER: a computational framework to evaluate readiness of datasets for machine learning

**DOI:** 10.1186/s12911-024-02544-w

**Published:** 2024-06-04

**Authors:** Meysam Ahangaran, Hanzhi Zhu, Ruihui Li, Lingkai Yin, Joseph Jang, Arnav P. Chaudhry, Lindsay A. Farrer, Rhoda Au, Vijaya B. Kolachalama

**Affiliations:** 1 Department of Medicine, Boston University Chobanian & Avedisian School of Medicine, Boston, MA USA; 2 Department of Neurology, Boston University Chobanian & Avedisian School of Medicine, Boston, MA USA; 3 Department Ophthalmology, Boston University Chobanian & Avedisian School of Medicine, Boston, MA USA; 4 Department of Epidemiology, Boston University School of Public Health, Boston, MA USA; 5 Department of Biostatistics, Boston University School of Public Health, Boston, MA USA; 6 Boston University Alzheimer’s Disease Research Center, Boston, MA USA; 7 The Framingham Heart Study, Boston University Chobanian & Avedisian School of Medicine, Boston, MA USA; 8 Department of Anatomy and Neurobiology, Boston University Chobanian & Avedisian School of Medicine, Boston, MA USA; 9 Department of Computer Science, Boston University, Boston, MA USA; 10 Faculty of Computing & Data Sciences, Boston University, Boston, MA 02215 USA

**Keywords:** Machine learning, Data readiness, Data quality measure, Feature engineering

## Abstract

**Background:**

Machine learning (ML) has emerged as the predominant computational paradigm for analyzing large-scale datasets across diverse domains. The assessment of dataset quality stands as a pivotal precursor to the successful deployment of ML models. In this study, we introduce DREAMER (**D**ata **REA**diness for **M**achin**E** learning **R**esearch), an algorithmic framework leveraging supervised and unsupervised machine learning techniques to autonomously evaluate the suitability of tabular datasets for ML model development. DREAMER is openly accessible as a tool on GitHub and Docker, facilitating its adoption and further refinement within the research community..

**Results:**

The proposed model in this study was applied to three distinct tabular datasets, resulting in notable enhancements in their quality with respect to readiness for ML tasks, as assessed through established data quality metrics. Our findings demonstrate the efficacy of the framework in substantially augmenting the original dataset quality, achieved through the elimination of extraneous features and rows. This refinement yielded improved accuracy across both supervised and unsupervised learning methodologies.

**Conclusion:**

Our software presents an automated framework for data readiness, aimed at enhancing the integrity of raw datasets to facilitate robust utilization within ML pipelines. Through our proposed framework, we streamline the original dataset, resulting in enhanced accuracy and efficiency within the associated ML algorithms.

**Supplementary Information:**

The online version contains supplementary material available at 10.1186/s12911-024-02544-w.

## Background

The proliferation of machine learning (ML)-based technologies across scientific and industrial domains underscores the importance of developing sophisticated and precise ML pipelines. Despite advancements in this area, there remains a dearth of comprehensive research aimed at assessing the readiness of data inputs to such pipelines, a crucial step towards constructing effective and broadly applicable models [1, 2]. Data scientists reportedly expend a significant portion of their efforts, approximately 80%, on iterative pre-processing tasks such as data cleansing, validation, and transformation prior to model construction. While data utilization varies among users and contexts, challenges related to data readiness persist across diverse stakeholders [3–5]. Given the data-intensive nature of most ML models, which often necessitate sizable datasets, the quality of the input data directly impacts the efficacy of models produced through various ML frameworks. Consequently, there has been a growing call within the data science community to assess dataset quality and ascertain its suitability for ML model development. Thus, investigating dataset readiness emerges as a critical research area, underscored by the pressing need for computational tools capable of systematically evaluating ML data readiness [6, 7].

Previous research has identified a range of metrics designed to instill user confidence in data integrity and facilitate interpretability across various dimensions of data quality. Notable among these metrics are Class Overlap, Label Purity, Class Parity, Feature Relevance, Data Homogeneity, Data Fairness, Correlation Detection, Data Completeness, Outlier Detection, and Data Duplicates (Supplementary Notes 1). Additionally, several tools have been proposed in prior literature to assess data readiness, including but not limited to AutoML [8], Datasheets [9], Data Statements [10], FactSheets [11], Dataset Nutrition Label [12], Model Cards [13], Datamaid [14], Codebook [15], IBM Data Quality Toolkit [16], and Data Readiness Report [17] (Supplementary Notes 2). While these tools offer considerable flexibility in evaluating data quality, their primary focus has not been on assessing data readiness in conjunction with enhancing the efficacy of subsequent ML model development using the same datasets. We contend that by integrating data readiness assessment with the downstream ML task, an opportunity arises to enhance the reliability of ML model research [18, 19].

The central premise of the proposed framework extends beyond the utilization of conventional data quality measures solely to elevate the readiness level of datasets for integration into ML pipelines. Instead, it leverages ML concepts to dynamically adjust data quality measures to suit the specific characteristics of each dataset. Unlike existing data readiness methodologies, the proposed framework adopts a dataset-dependent approach, wherein the significance of individual data quality metrics is tailored to the intrinsic attributes of the corresponding dataset. In this study, both supervised and unsupervised approaches are employed to delineate the relative importance of each data quality metric within a given dataset.

### Implementation

**Fig. 1:**
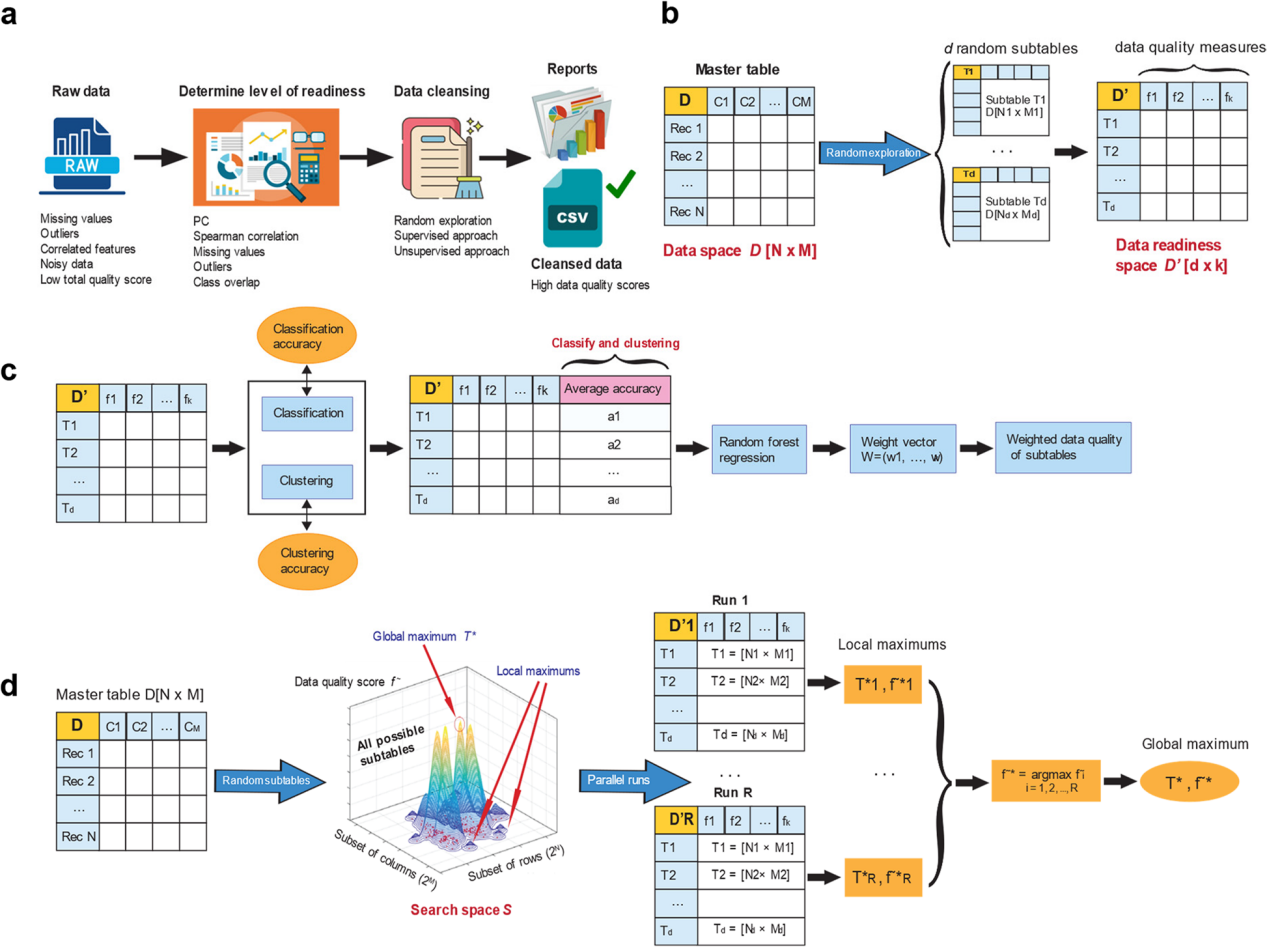
DREAMER framework. **a** The DREAMER architecture workflow delineates the process for evaluating the readiness of a tabular dataset for machine learning. Input to DREAMER comprises the tabular dataset under scrutiny, which undergoes a sequence of automated procedures, culminating in the generation of a structured tabular dataset conducive to machine learning analysis. **b** The transformation of the data space *D* into data readiness space *D’* involves constructing a new dataset from the master dataset. The master dataset dimension is denoted as *N×M*, while the data readiness dataset assumes dimensions of *d×k*, where *d* represents the number of random sub-tables and *k* indicates the number of data quality measures. **c** The process involves learning the weights of data quality measures from dataset *D’* utilizing regression methodology. The average accuracy of clustering and classification serves as the target value for the regression algorithm. Subsequently, weighted total quality of sub-tables is computed post-weight learning to ascertain the best sub-table boasting the highest data quality. **d** The search space of DREAMER scales proportionally with the size of the master dataset (both in terms of rows and columns). We execute DREAMER *R* times to identify the best sub-table of each run as local maximum, subsequently selecting the sub-table exhibiting the highest data quality as a potential global maximum

In this study, we present a computational framework designed to facilitate automated assessment of data readiness for machine learning applications (Fig. 1). While various methodologies exist for constructing such frameworks, we have developed a framework that evaluates data readiness by integrating a spectrum of quality metrics with the dataset’s capacity for precise classification and clustering. Termed DREAMER ((**D**ata **REA**diness for **M**achin**E** learning **R**esearch)), our framework is introduced as an open-source tool for researchers, representing its initial version. To evaluate its efficacy, we applied DREAMER to assess tabularly curated data sourced from three prominent datasets: the Alzheimer’s Disease Neuroimaging Initiative (ADNI) [20], the Framingham Heart Study (FHS) [21], and Wisconsin Diagnosis Breast Cancer (WDBC) [22]. Our analysis demonstrates that DREAMER effectively evaluates ML readiness across these datasets, elucidating the combination of person-level features and instances conducive to optimal ML-driven outcomes. Notably, the original data for each dataset was sourced directly from the respective cohorts in tabular format.

Given our framework’s focus on assessing readiness with a view to enhancing ML performance, each dataset (referred to as the master table *D*) comprises a variety of input features columns alongside a single column dedicated to the output label of interest. Our automated framework takes the master table *D*, composed of *M* columns (inclusive of the specified output label column) and *N* rows, as input for assessing its readiness for ML tasks. A key aspect of our strategy involves the identification of a sub-table T* within *D* boasting the highest quality score (represented by *f̃*). This approach entails random sampling of a set of sub-tables *T*_*i*_ [*N*_*i*_, *M*_*i*_], followed by the computation of quality metrics for each.

Supervised and unsupervised algorithms are subsequently trained on each sub-table, utilizing *N*_*i*_ rows, *M*_*i*_ features and the output label, with the average accuracy derived from the algorithms forming a discrete space *S*. The optimization objective revolves around exploring this space to pinpoint T* with the highest quality. Notably, the search space *S* is vast, encompassing 2^*N*^ × 2^*M*^ points, thereby rendering the search strategy akin to a subset sum problem [23], recognized for its *NP*-Complete nature and intractable exponential runtime. Consequently, identifying an optimal sub-table emerges as a computationally daunting task necessitating the development of efficient algorithms and optimization strategies. Our proposed DREAMER framework represents an empirical approach to identify T*, with results obtained from the FHS, ADNI, and WDBC datasets serving as a proof-of-principle for the efficacy of our automated framework in evaluating their ML readiness.

### Study population

Clinical, genetic, demographic, and neuropsychological assessments, along with functional evaluations, were acquired from ADNI and FHS datasets. Additionally, digitized images were examined from WDBC dataset. These datasets were subsequently processed and organized into tabular formats, as depicted in Table 1. The ADNI study, a longitudinal multicenter endeavor, is dedicated to the development of biomarkers aimed at early detection and monitoring of Alzheimer’s disease. The FHS, a longitudinal community cohort investigation spanning multiple generations, has amassed a comprehensive repository of data concerning cardiovascular health, with subsequent expansions to investigate factors influencing cognitive decline, dementia, and Alzheimer’s disease. The WDBC dataset comprises numerical data derived from digitized fine needle aspirate (FNA) images of breast masses, specifically gathered for breast cancer diagnosis purposes.

**Table 1:** Study population of FHS, ADNI, and WDBC datasets and their characteristics

Dataset	FHS (*n* = 5209)		ADNI (*n* = 2376)					WDBC (*n* = 569)	
Classification feature	Probable dementia present		Dementia diagnosis at baseline					Breast cancer diagnosis	
Labels characteristic	No Dementia (*n* = 1245)	Probable Dementia (*n* = 1220)	CN (*n* = 534)	LMCI (*n* = 672)	EMCI (*n* = 411)	SMC (*n* = 325)	AD (*n* = 407)	Benign (not cancerous) (*n* = 357)	Malignant (cancerous) (*n* = 212)
Age	44.3 ± 8.3 [29, 62]	43 ± 8 [29, 62]	73.4 ± 6.2 [55, 89]	73.7 ± 7.5 [54, 91]	71.2 ± 7.4 [55, 89]	71 ± 6.4 [56, 90]	74.8 ± 7.9 [55, 90]		
Gender, male (%)	607 (48.7%)	384 (31.4%)	252 (47.1%)	411 (61.1%)	227 (55.2%)	126 (38.7%)	230 (56.5%)		
Education	4.9 ± 2.9 [0, 10]	5 ± 2.3 [0, 10]	16.4 ± 2.6 [6, 20]	15.9 ± 2.8 [4, 20]	16 ± 2.6 [10, 20]	16.7 ± 2.3 [8, 20]	15.1 ± 2.9 [4, 20]		
Data quality scores	PC = 0.7674 Spearman correlation = 0.7363 Missing values = 0.2689 Outliers = 1.0 Class overlap = 0.8071 Total weighted quality = 0.6481		PC = 0.7466 Spearman correlation = 0.7312 Missing values = 0.7497 Outliers = 0.1347 Class overlap = 0.1654 Total weighted quality = 0.4656					PC = 0.6052 Spearman correlation = 0.5782 Missing values = 1 Outliers = 0.0875 Class overlap = 0.9315 Total weighted quality = 0.6335	
Classification / Clustering accuracy	Classification accuracy = 0.8594 Clustering accuracy = 0.4332		Classification accuracy = 0.5134 Clustering accuracy = 0.6012					Classification accuracy = 0.4804 Clustering accuracy = 0.5792	
Number of features	81		45					30	

In our study, we utilized data from the ADNI dataset, specifically focusing on the ADNIMERGE table comprising 2,376 participants and 52 features. These features encompassed 44 baseline factors, six demographic features, one genetic feature (APOE4), and one diagnosis class feature at baseline (DX_bl). The DX_bl feature encompassed five categories—cognitively normal (CN), late mild cognitive impairment (LMCI), early mild cognitive impairment (EMCI), significant memory concern (SMC), and Alzheimer’s disease (AD)—reflecting diagnosis groups at baseline. Our experiment incorporated all 44 baseline features, alongside participant education status (PTEDUCAT) and the DX_bl class feature, totaling 46 columns of data. Furthermore, the Framingham Heart Study (FHS) dataset comprised 5,209 rows and 82 columns, encompassing demographic information, anthropometric measurements, smoking status, blood test results, neuropsychological assessments, and verified outcomes of dementia. All features were collected statically during participants’ initial visits, with the outcome represented by a binary measure indicating probable dementia presence. Lastly, the Wisconsin Diagnosis Breast Cancer (WDBC) dataset included 569 participants and 30 features, with no missing values. All features were numerical, with the class feature “Diagnosis” representing a categorical variable with two values: “Benign” indicating non-cancerous conditions and “Malignant” indicating cancerous conditions (Supplementary Fig. 1).

### Computational framework

Our study applied the proposed model to the ADNI, FHS, and WDBC datasets, with the objective of deriving the most refined table characterized by optimal data quality achieved through the exclusion of less pertinent rows and columns. The algorithm was executed ten times, varying the number of sub-tables from 1000 to one million, to iteratively identify the most cleansed dataset. The resultant cleansed dataset exhibits the capability to effectively differentiate individuals based on diagnostic groups—specifically, five diagnosis groups in the ADNI dataset (CN, LMCI, EMCI, SMC, and AD), two diagnosis groups in the FHS dataset (Probable Dementia and No Dementia), and two diagnosis groups in the WDBC dataset (Cancerous and Not Cancerous). Consequently, this refined dataset stands poised for utilization in AI tasks aimed at knowledge discovery and prediction, leveraging both supervised and unsupervised approaches.

Within the realm of data readiness, we delineated five key data quality measures: Pearson Correlation (*PC*), Spearman Correlation, Missing Values, Outliers, and Class Overlap. All measures underwent normalization within the range [0, 1], with values nearing one signifying heightened data quality within a sub-table. Thresholds for the RER and CER were established at 0.2 and 0.5, respectively. Consequently, the proposed algorithm is poised to exclude a maximum of 20% of rows and 50% of columns from datasets to generate random sub-tables. Properties of each run’s sub-tables are preserved as individual CSV files, with specification of the optimal sub-table exhibiting the highest $\widetilde f$ score saved in a separate CSV file. Furthermore, a TXT file captures the details of the top-performing CSV sub-table among all runs, including the indices of selected columns and rows from the master dataset. This CSV file represents the refined version of the master dataset, indicative of the global optimum point, encapsulating the best sub-table T* with the highest total quality score $\widetilde f$  f̃*.

Given a raw dataset *D* [*N×M*] comprising *N* rows and *M* columns denoted as *c*_*1*_, *c*_*2*_, *…, c*_*M*_, we derive d random sub-tables, *T*_*i*_, each comprising *N*_*i*_ rows and *M*_*i*_ columns such that *∀i N*_*i*_ *⊆ N* and *M*_*i*_ *⊆ M*. The selection of rows and columns within these sub-tables follows a uniform distribution. Subsequently, we compute *k* data quality metrics denoted as *F = {f*_*1*_, *f*_*2*_, *…, f*_*k*_ *}* for all sub-tables. Based on this, we define *d* sub-table instances and *k* corresponding quality metrics, thereby delineating the data readiness space as *D’[d×k]*. The primary objective is to identify the optimal sub-table T*[N*×M*] within the original dataset *D*, characterized by the highest level of data quality denoted by $\widetilde f$. The value of data quality $\widetilde f$ is derived from *k* data quality measures within the data readiness space *D’*, expressed as:

$$\widetilde f=\sum\nolimits_{i=1}^kw_if_i$$

where data quality measure is represented by *f*_*i*_, each assigned a weight denoted by *w*_*i*_. Within each iteration, classification and clustering algorithms are implemented on the respective sub-tables, employing a designated feature as the class label. Subsequently, classification accuracy is determined utilizing the 10-fold Cross Validation method across all *d* sub-tables. This process yields a new dataset comprising *d* samples, each representing a distinct sub-table, incorporating *k* data quality features alongside the average classification and clustering accuracies. Random forest regression analysis is then applied to ascertain the weight vector *W = (w*_*1*_, *…, w*_*k*_ *)* for features *F = (f*_*1*_, *…, f*_*k*_ *)*. Finally, the mean value of all *R* weight vectors across iterations, denoted as W* = mean (W_*1*_, *…, W*_*R*_ *)*, serves as the weights for the data quality features *F* in computing the value of $\widetilde f$ for the sub-tables.

Within the data readiness space *D’*, each sub-table *T*_*i*_ *[N*_*i*_, *M*_*i*_ *]* represents a subset of rows and columns derived from the original dataset *D*. Our objective is to identify the sub-table T* within *D’* exhibiting the highest $\widetilde f$ value, constituting an optimization challenge within the search space *S* in *D’*. This task involves navigating a discrete search space comprising 2^*N*^ × 2^*M*^ points, with each point associated with a corresponding $\widetilde f$ value. The optimization strategy is directed towards pinpointing the point T* boasting the highest$\widetilde f$ score. Notably, this optimization problem mirrors the subset sum problem, recognized as an *NP*-Complete problem characterized by intractable exponential running time.

To comprehensively explore the search space *S*, an effective heuristic approach is essential to streamline the search process and identify the global optimum of *S* in a pragmatic manner. In pursuit of this objective, we implement the random restart strategy, wherein, during each iteration, *d* sub-tables are randomly selected from the original dataset *D*, encompassing a randomized subset of rows and columns. To mitigate the risks of overfitting and underfitting inherent in machine learning algorithms, we define maximum threshold values for the Columns Exclusion Ratio (*CER*) and Rows Exclusion Ratio (*RER*). These thresholds dictate that the proportion of columns and rows excluded from *D* to construct sub-tables within *D’* should not surpass the specified *CER* and *RER* thresholds, respectively. Notably, the rows of *D’* correspond to sub-tables *T*_*i*_ *[N*_*i*_ *×M*_*i*_ *]* extracted from the primary dataset *D[N×M]*, while the columns encompass the data quality features *F = {f*_*1*_, *f*_*2*_, *…, f*_*k*_ *}*, alongside the total quality score $\widetilde f$. The algorithm is executed *R* times, employing a randomized subset selection strategy to ensure thorough exploration of the search space.

Each run is executed on an independent CPU core utilizing the Python multiprocessing pool method, enabling the full utilization of multiple processors available on the system. Leveraging the pooling method facilitates the parallel execution of *R* runs, effectively distributing the input data across concurrent processes. The optimum point of the *i*th run, denoted as ${T}_{i}^{*}$, along with its corresponding total quality score ${\widetilde f}_i$, is identified. Through *R* iterations, the dataset *D* is transformed into the data readiness space *D’*, with each run selecting the optimal sub-table. Ultimately, the best optimum point T* is chosen from among all *R* optimum points {${T}_{1}^{*}$, ${T}_{2}^{*}$, …, ${T}_{R}^{*}$}, representing the global optimum point with the highest total quality score $\widetilde f$*. The pseudocode outlining the proposed algorithm is presented below as **Algorithm 1,** where the input is the dataset *D* serving as the master dataset, and the output is a sub-table T* exhibiting the highest data quality, representing the cleansed version of *D*.

**Figure:**
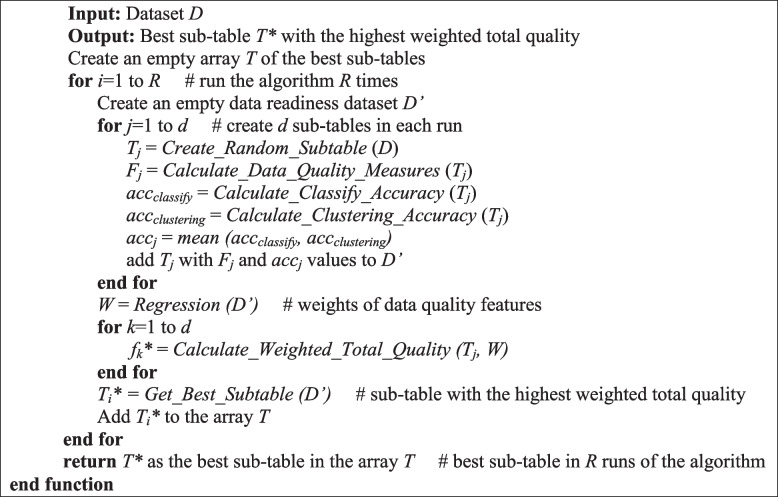
**Algorithm 1.** DREAMER v1.0 (Dataset *D*)

### Quality metrics

A comprehensive set of established quality metrics was computed for each sub-table derived from the original dataset, subsequently serving as features indicative of the data readiness space. These metrics encompass Average Pearson Correlation (*PC*), Average Spearman Correlation, Missing Values, Outliers, and Class Overlap. The average *PC* measure is derived by computing the mean value of the *PC* coefficients across all feature pairs within the corresponding subset of features. The *PC* measure is defined within the range [-1, + 1] and is calculated as follows:

$$PC= \frac{\sum ({x}_{i}-\stackrel{-}{x })({y}_{i}-\stackrel{-}{y })}{\sqrt{\sum {\left({x}_{i}- \stackrel{-}{x}\right)}^{2}\sum {({y}_{i}-\stackrel{-}{y})}^{2}}}$$

We regarded 1 – *PC* as a data quality metric for each sub-table. In quantifying the prevalence of missing values within a sub-table, we computed the Missing Values (*MV*) as the proportion of missing values, utilizing 1 – *MV* as an indicator of data quality. The *PC* score denotes a linear correlation among features. To evaluate the presence of non-linear correlations among features, we employed the average *Spearman Correlation* (*ρ*) measure, calculated according to the following formula:

$$\rho =1- \frac{6\sum {d}_{i}^{2}}{n({n}^{2}-1)}$$

In the aforementioned formula, *d*_*i*_ represents the disparity between the two ranks within each sample, with *n* denoting the total number of samples in the dataset. We adopt the score 1 – *ρ* as a data quality metric for a given sub-table. To quantify the presence of outliers within a dataset, we computed the *Median Absolute Deviation* (*MAD*), leveraging a univariate approach to outlier detection. The test statistic for the *MAD* method is computed analogously to the *z-score* method, as illustrated by the following formula:

$$MAD\;=\;median\;\left(\left|x_i-median\left(X\right)\right|\right)$$

For each data point *x*_*i*_, if the absolute value of $\frac{{x}_{i}-median\left(X\right)}{MAD}$ exceeds 3, it is identified as an outlier. Consistent with prior methodology, we employed 1 – *MAD* as the data quality metric for each sub-table. The *MAD* method exhibits greater robustness compared to the *z-score* method due to its reduced sensitivity to outlier influence; outliers exert a diminished impact on the median relative to the mean. Conversely, the *z-score* method, reliant on mean and standard deviation, is significantly influenced by outliers. Thus, the *MAD* method represents a robust approach for detecting outlier data within non-normally distributed datasets.

The Class Overlap measure is determined by the R value, as proposed by Oh [24], predicated on the premise that a sample from a class *C*_*l*_ is considered to be overlapped with other samples if the count of samples within its *k* nearest neighbors (*kNN*) that pertain to a class other than *C*_*l*_ exceeds a predefined threshold. The *R* value is computed using the following formula:

$$\mathrm R\;=\;\frac1N{\textstyle\sum_{l=1}^n}{\textstyle\sum_{i=1}^{\left|C_l\right|}}\;\lambda\left(\left|kNN\;\left(x_i^1,\;D\;-\;C_l\right)\right|-\theta\right)$$

In the provided formula, *D* represents the dataset comprising *N* samples distributed among *n* classes denoted as *C*_*1*_, *C*_*2*_, *…, C*_*n*_. The variable ${x}_{i}^{l}$ denotes the *i*th sample within the class *C*_*l*_. The set *kNN*(${x}_{i}^{l}, D-{C}_{l}$) refers to the collection of samples in the *k* nearest neighbors of ${x}_{i}^{l}$ that belong to classes other than *C*_*l*_. The function *λ*(*x*) operates as a binary function, returning 1 when *x* > 0 and 0 otherwise. The threshold value *θ* is confined to the interval [0, *k*/2]. The time complexity for calculating the *R* value across *N* samples is *O* (*N* ^2^). The *R* value ranges from 0 to 1, with the value 1-*R* serving as a data quality metric for a given sub-table. Within this study, parameter settings for *k* and *θ* are designated as *k* = 7 and *θ* = 3, respectively. With these parameters, a sample is deemed to be within the overlapping region if at least four of its seven nearest neighbors belong to a different class.

In the context of computing correlation, identifying outliers, and assessing class overlap, necessitating numerical values, we excluded five non-numerical features, along with the genetic feature APOE4, from the 52 static features within the ADNI dataset for sub-table construction. Specifically, the omitted features encompass AGE, PTGENDER, PTETHCAT, PTRACCAT, PTMARRY, and APOE4. For the generation of random sub-tables, we considered 45 static features of the ADNI dataset and utilized the DX_bl variable as the class feature. Similarly, within the FHS dataset, 81 features were utilized alongside the DEMRV046 class feature for generating random sub-tables. Lastly, for the WDBC dataset, we considered all 30 features, as they are numerical, and utilized the class feature DIAGNOSIS to generate random sub-tables. Although the total number of subsets across the ADNI, FHS, and WDBC datasets amounts to 2^45^, 2^81^, and 2^30^ respectively, our exploration was confined to a limited region of the comprehensive search space through the adoption of a random restart strategy. Moreover, by selecting random rows from the ADNI, FHS, and WDBC tables, which respectively comprise 2,376, 5,209, and 569 rows, the scale of this problem’s total search space escalates to an intractable magnitude exceeding 2^2376^, 2^5209^, and 2^569^ unique states.

To ascertain the weights of four data quality measures, we employed *Random Forest* (*RF*) with 20 estimators and *Stochastic Gradient Descent* (*SGD*) classifiers, utilizing a 10-fold cross-validation to compute the accuracy of each sub-table classification within each run. In the realm of unsupervised learning, we leveraged agglomerative and *k*-means clustering algorithms, evaluating the accuracy of clustering for each sub-table through the computation of the *Silhouette Coefficient S*, determined by the following formula:

$$S= \frac{b-a}{\text{m}\text{a}\text{x}(a,b)}$$

In the aforementioned equation, *a* represents the mean distance between a sample and all other samples within the same class, while *b* denotes the mean distance between a sample and all other samples in the next nearest cluster. The average Silhouette Coefficient (*S*) value obtained from the two clustering algorithms signifies the average accuracy of clustering for the respective sub-table. A higher Silhouette coefficient indicates more well-defined clusters, with scores ranging between − 1 for incorrect clustering and + 1 for highly dense clustering. Scores near zero suggest overlapping clusters, while values within the interval [0, 1] are considered as the accuracy measure of the clustering algorithm. We treat Silhouette Coefficient values within the interval [-1, 0] as zero. Ultimately, the average accuracy of both classification and clustering algorithms, weighted equally, serves as the accuracy metric for the associated sub-table. We utilized *Random Forest Regression* with 20 estimators to derive the weight vector for data quality measures, with the accuracy of each sub-table serving as the target value for regression.

DREAMER has been made publicly available as a web-based utility, catering to researchers keen on assessing the readiness of their tabular datasets (referred to as the master table). Upon authentication via our integrated user management system, researchers can securely upload their data in CSV format and receive a comprehensive report outlining DREAMER’s analysis of the master table’s readiness for ML applications. Additionally, users will receive actionable recommendations, including an ML-ready dataset, typically derived as a subset of the master table optimized for ML tasks. All computational processes associated with these functionalities are executed on a high-performance computing cluster (SCC) situated at the Massachusetts Green High Performance Computing Center. Upon completion of the computation, users will be promptly notified via email and provided with the option to download both the refined dataset and the quality assessment report.

### Web development framework

**Fig. 2:**
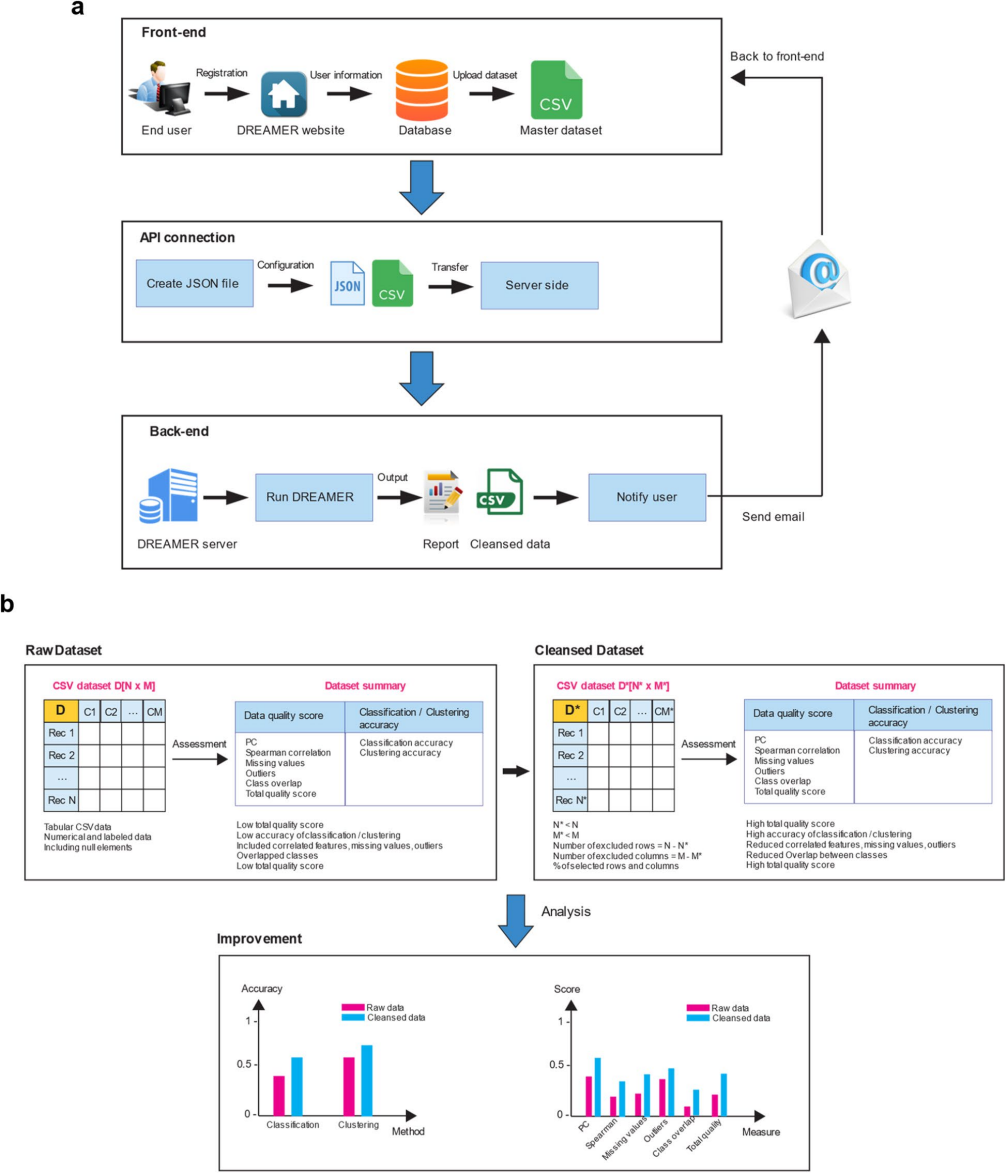
Architecture of the DREAMER web framework. **a** DREAMER comprises three primary components: the front-end, API connection, and back-end. Within the front-end interface, users register and subsequently upload a raw CSV dataset file to the website. The API connection stage involves the generation of a JSON configuration file corresponding to the uploaded dataset, encompassing DREAMER parameters. This JSON file, along with the master dataset, is then transmitted to the server. On the back-end, the principal DREAMER process operates on the master dataset, resulting in the generation of a cleansed CSV file accompanied by various reports and statistical analyses. Upon completion of the DREAMER process, users receive email notifications and can access the cleansed dataset and reports within their profile section on the website. **b** DREAMER enhances the quality of raw datasets by elevating data quality scores and improving the accuracy of classification and clustering algorithms. It selectively removes correlated features and rows from the original dataset to enhance the overall quality score of the cleansed dataset

We have developed DREAMER as a web-based tool (https://github.com/vkola-lab/DREAMER), which facilitates users in registering on our platform, uploading their datasets, assessing their machine learning readiness, and obtaining cleansed datasets as outputs. The intuitive user interface accommodates the uploading of a master CSV dataset. Upon initiation, this action initiates an Application Programming Interface (API) connection, generating a JSON configuration file containing DREAMER parameters pertinent to the master dataset, subsequently transmitting it to our server. The backend system then assumes control, executing the core DREAMER processes on the dataset, resulting in a sanitized CSV file accompanied by comprehensive reports and statistical analyses. Upon conclusion of the DREAMER procedures, users are promptly notified via email and granted access to download the complete package, inclusive of the cleansed dataset, reports, and data readiness metrics (Fig. 2).

## Results

The algorithm underwent ten iterations, each involving the analysis of randomly selected sub-tables ranging in size from 1000 to one million, to evaluate the efficacy of the proposed model across an expanded search space. The mean weight vectors derived from ten iterations on the ADNI, FHS, and WDBC datasets are as follows: W*_ADNI_ = (0.1606, 0.1573, 0.2021, 0.0094, 0.4703), W*_FHS_ = (0.2069, 0.1994, 0.2626, 0.0241, 0.3069), and W*_WDBC_ = (0.1917, 0.1911, 0, 0.1990, 0.4183) for the attributes *PC*, *Spearman Correlation*, *Missing Values*, *Outliers*, and *Class Overlap*, respectively. Consequently, among the five data quality measures evaluated across all three datasets, *Class Overlap* emerged as the most significant measure in the ADNI, FHS, and WDBC datasets. The mean ranges of all data quality features across the three datasets are presented in Supplementary Table 1, accompanied by 95% confidence intervals derived from the assumption of a normal distribution.

 Supplementary Table 2 displays the best sub-tables from each run for the ADNI, FHS, and WDBC datasets. The sub-table with the highest data quality for the ADNI dataset is derived from Run 10, with a total quality score of $\widetilde f$^*^ = 0.6662. This sub-table consists of 2,118 rows and 23 columns, selected from an initial master table of 2,376 rows and 45 columns, indicating an increase in data quality of 0.2006 compared to the ADNI master table’s quality score of 0.4656. A similar improvement was observed in the FHS dataset, where the best sub-table also came from Run 10, achieving a total quality score of $\widetilde f$^*^ = 0.7232. This sub-table comprises 4,354 rows and 42 columns, extracted from an original dataset of 5,209 rows and 81 columns, demonstrating an increase in data quality of 0.0751 compared to the FHS master table’s score of 0.6481. In the WDBC dataset, the optimal sub-table was obtained from Run 4, with a total quality score of $\widetilde f$^*^ = 0.843. This sub-table contains 455 rows and 15 columns, compared to the original table’s 569 rows and 30 columns, yielding a significant improvement in data quality of 0.2095 over the WDBC master table, which had a score of 0.6335.

**Fig. 3:**
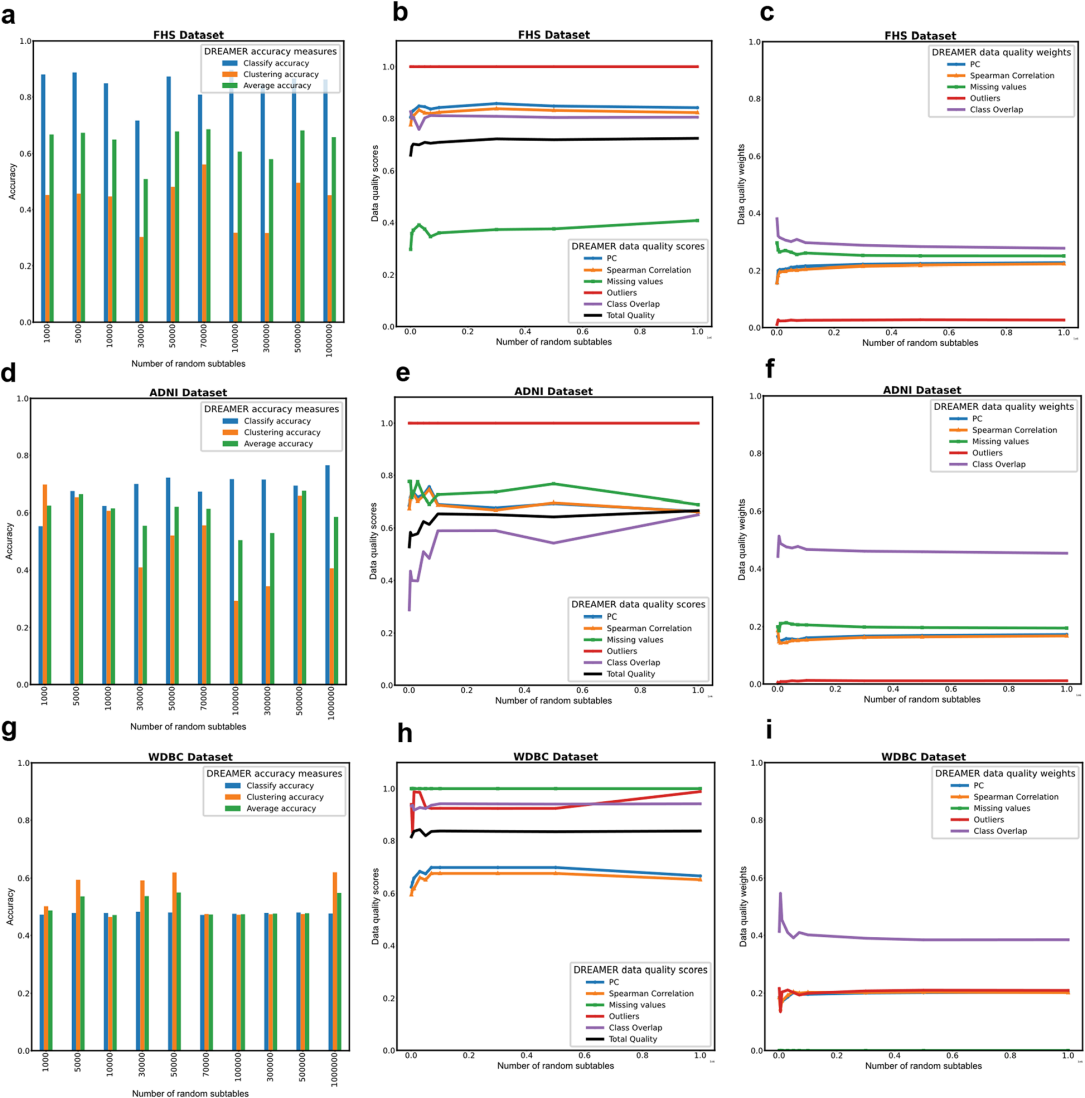
Convergence analysis of the DREAMER framework across multiple datasets. **a** Clustering and classification analysis in the FHS dataset as a function of the number of random sub-tables. **b** Plot showing the relationship between data quality scores and the number of random sub-tables in the FHS dataset. **c** Diagram illustrating the relationship between data quality weights and the number of random sub-tables in the FHS dataset. **d** Clustering and classification analysis in the ADNI dataset as a function of the number of random sub-tables. **e** Plot showing the relationship between data quality scores and the number of random sub-tables in the ADNI dataset. **f** Diagram illustrating the relationship between data quality weights and the number of random sub-tables in the ADNI dataset. **g** Clustering and classification analysis in the WDBC dataset as a function of the number of random sub-tables. **h** Plot showing the relationship between data quality scores and the number of random sub-tables in the WDBC dataset. **i** Diagram illustrating the relationship between data quality weights and the number of random sub-tables in the WDBC dataset

Experimental outcomes from ten iterations of the DREAMER algorithm on the FHS, ADNI, and WDBC datasets are depicted in Fig. 3. The results for the FHS dataset indicate variability in both classification and clustering accuracy as the number of sub-tables increased. In contrast, the ADNI dataset exhibited a slight improvement in classification accuracy with more random sub-tables, while clustering accuracy showed fluctuations as the number of sub-tables increased. For the WDBC dataset, clustering accuracy also demonstrated variability with the number of sub-tables, whereas classification accuracy remained relatively stable despite increasing the number of sub-tables.

Our findings further reveal that in all three datasets, the weights and scores for data quality measures, as well as the total quality of the optimal sub-table, reached a steady state after generating approximately 100,000 sub-tables. Additionally, the data suggest that the total data quality score of the best sub-tables generally tends to increase as the number of sub-tables grows, indicating that DREAMER can enhance the quality of the master dataset by exploring a broader range within the search space.

In the FHS dataset, the number of random sub-tables showed a positive correlation with *PC*, *Spearman correlation*, *Missing values* scores, and the total quality of the optimal sub-table. However, this measure did not exhibit significant correlation with other data quality metrics, nor with classification and clustering accuracy. The number of random sub-tables also demonstrated a positive correlation with weights for *PC*, *Spearman correlation*, and *Outliers*, while exhibiting a negative correlation with weights for *Missing values* and *Class overlap* (Supplementary Fig. 2a, b).

In the ADNI dataset, the number of random sub-tables correlated positively with classification accuracy but negatively with clustering accuracy. Additionally, a positive correlation was observed with *Class overlap* score and the total quality of the best sub-table, whereas negative correlations were noted with *PC* and *Spearman correlation* scores, with a slight negative correlation with *Missing values*. Regarding the weights of data quality measures, the number of random sub-tables had a positive correlation with weights for *PC*, *Spearman correlation*, and *Outliers*, but a negative correlation with weights for *Missing values* and *Class overlap* (Supplementary Fig. 2c, d).

For the WDBC dataset, the number of random sub-tables exhibited a positive correlation with scores for *PC*, *Spearman correlation*, *Outliers*, *Class overlap*, clustering accuracy, and the total quality of the best sub-table, but showed no significant correlation with classification accuracy. The number of random sub-tables was also positively correlated with weights for *PC*, *Spearman correlation*, and *Outliers*, but negatively correlated with the weight for *Class overlap* (Supplementary Fig. 2e, f). Supplementary Fig. 3 provides a regression analysis of the DREAMER framework on the FHS, ADNI, and WDBC datasets across ten iterations.

To establish a practical baseline, we used the average precision of clustering and classification tasks as our primary metric, allowing us to determine weights for the data quality measures and assess overall data readiness. Within this framework, we posit that a high-quality dataset is characterized by clear cluster separation and distinct sample discrimination. The toolbox also provides a range of customizable features, enabling users to set their own objectives for identifying high-quality datasets that align with their criteria. This user-driven flexibility promotes the discovery of insights across various tabular datasets, highlighting the versatility and adaptability of our proposed approach.

**Fig. 4:**
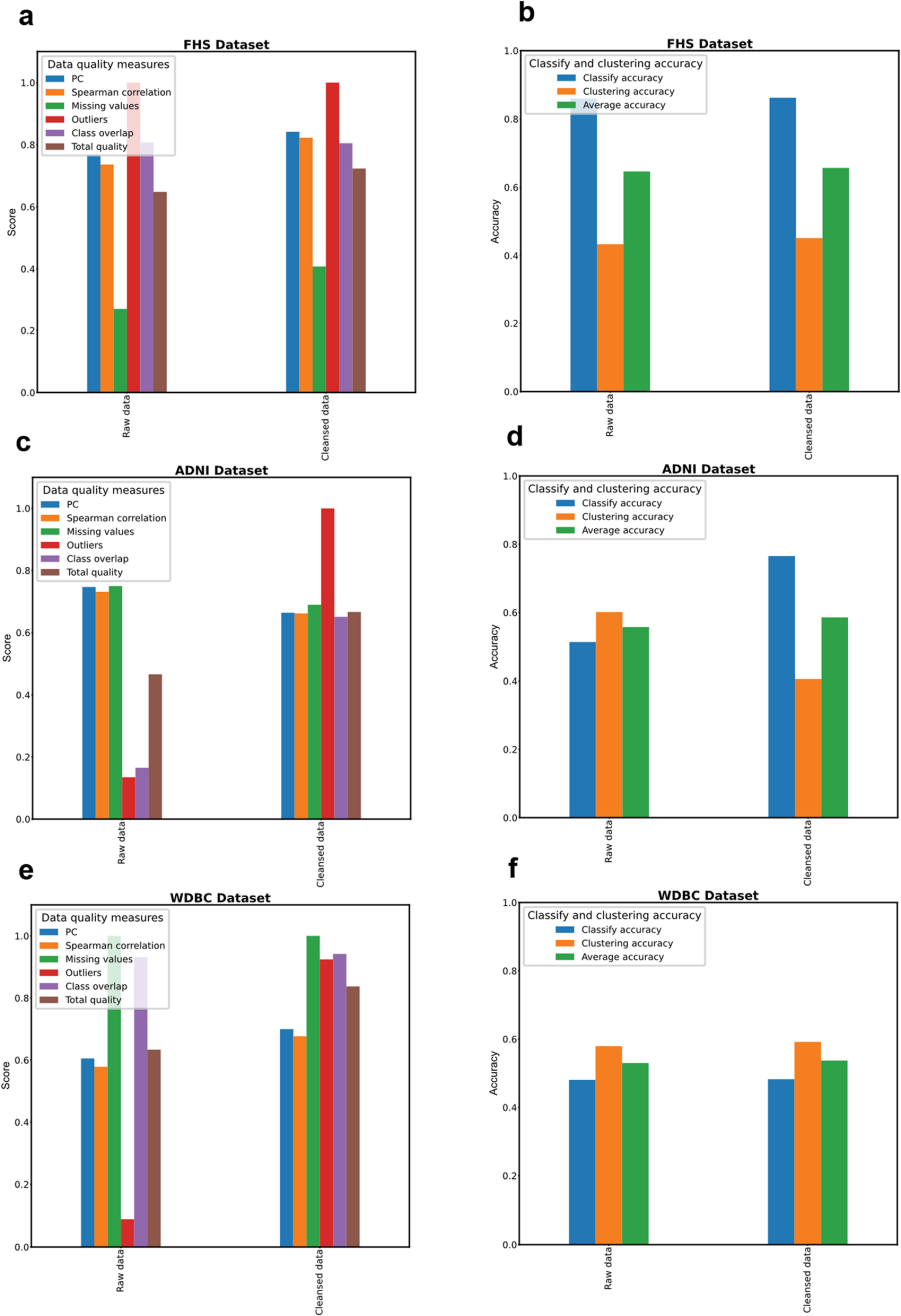
DREAMER framework evaluation across multiple datasets. **a** Comparison of raw and cleansed data quality scores for the FHS dataset, illustrating the impact of DREAMER’s data cleansing. **b** Comparison of classification and clustering accuracies between raw and cleansed data for the FHS dataset, providing insights into the impact of data cleansing on these metrics. **c** Comparison of raw and cleansed data quality scores for the ADNI dataset, illustrating the impact of DREAMER’s data cleansing. **d** Comparison of classification and clustering accuracies between raw and cleansed data for the ADNI dataset, providing insights into the impact of data cleansing on these metrics. **e** Comparison of raw and cleansed data quality scores for the WDBC dataset, illustrating the impact of DREAMER’s data cleansing. **f** Comparison of classification and clustering accuracies between raw and cleansed data for the WDBC dataset, providing insights into the impact of data cleansing on these metrics

Our results demonstrated that the total quality of cleansed datasets significantly improved across all three datasets examined. For the FHS and WDBC datasets, the cleansed data showed enhanced accuracy in both classification and clustering compared to the original raw data. In the ADNI dataset, while classification accuracy improved with data cleansing, clustering accuracy experienced a slight decline relative to the raw data (Fig. 4). This variability in outcomes underscores the importance of context and dataset-specific factors when evaluating the effectiveness of data quality enhancements.

## Discussion

Our study acknowledges several limitations. First, the data readiness space was constructed based on a limited set of well-established quality measures, and our framework focused exclusively on tabular datasets while targeting average accuracy in classification and clustering tasks. Despite these constraints, this approach effectively demonstrated an automated methodology for assessing a dataset’s readiness for ML applications. It is important to note that the complexity of the problem escalates with the broadening of the data readiness space. Additionally, because our framework concentrated on tabular datasets, its generalizability to other data types, such as images and text, may be limited. Computational demands also represent a significant challenge within our proposed framework; however, our experiments suggest that enhanced computational resources can lead to improved data quality.

In our framework, each point within the state space represents a random sub-table generated by selectively omitting certain rows and columns from the original dataset. While it is generally infeasible to examine the entire state space, the dataset with the highest quality can be identified by generating additional sub-tables. Two key parameters, *RER* and *CER*, govern which regions of the search space can be explored. Increasing the values of these parameters can significantly extend the algorithm’s runtime. It is important to note that the weights assigned to data quality features vary with each dataset, meaning that the specific weight values derived from our study might differ when applied to other datasets.

In this study, we designed a toolbox to automate the evaluation of data readiness in tabular datasets for machine learning applications. Our framework can be likened to a knowledge discovery process, where a structured dataset was transformed into a data readiness space using a defined set of quality measures. Through a systematic exploration of this data readiness space, we identified high-quality sub-datasets capable of producing high-performing machine learning models. Notably, the search space for this problem is exceedingly large, with full exploration limited by computational resources. Our results indicate that by expanding the search space through the generation of additional random sub-tables, it is possible to create a cleansed dataset with higher data quality. Experiments on the ADNI, FHS, and WDBC datasets revealed that increasing the number of random sub-tables from one thousand to one million led to an improvement in the total quality of the best sub-tables, suggesting that DREAMER moves toward a global optimum, representing the optimal cleansed dataset. This finding underscores that the DREAMER framework can enhance the quality of raw datasets, with the degree of improvement contingent on the computational resources available.

## Conclusions

The experimental results indicate that DREAMER effectively identifies low-quality features and records, leading to improved data quality through cleansing. This process creates more reliable data for ML pipelines. By applying DREAMER prior to ML pipeline execution, the accuracy of both classification and clustering tasks can be enhanced by eliminating noisy records and irrelevant features. Furthermore, reducing the master dataset by removing non-essential rows and columns results in increased computational speed for the ML algorithm. Thus, DREAMER not only enhances the accuracy of ML tasks but also improves the efficiency of the corresponding ML algorithms through data simplification. We designed DREAMER as a flexible tool with the potential for future extension, accommodating additional data quality measures, model performance metrics, and non-tabular data types.

## Availability and requirements

### Computational hardware and software

Python (version 3.9) was used for software development and plots were generated using *matplotlib* (version 3.4.3) and *seaborn* (version 0.11.2). *NumPy* (version 1.20.3) was used for vectorized numerical computation. Other Python libraries used to support data analysis include *pandas* (version 1.3.4), *scipy* (version 1.7.1), and *scikit-learn* (version 0.24.2). For model runs, we used the Python multi-processing pool method. The infrastructure to perform these computations was provided by Boston University Shared Computing Cluster (*SCC*). Each model run was performed using 28 high-speed CPU cores.

### Data availability

The ADNI and WDBC datasets are accessible through publicly available resources. The FHS dataset can be obtained upon request, subject to institutional approval.

### Model availability

More information on the web-based tool can be found on GitHub (https://github.com/vkola-lab/DREAMER). Additionally, a Docker container for DREAMER is available at https://hub.docker.com/repository/docker/ahangar/dreamer_docker.

### Code availability

Python scripts and user manuals are made available on GitHub (https://github.com/vkola-lab/DREAMER).

### Supplementary Information

Supplementary Material 1.

Supplementary Material 2.

Supplementary Material 3.

## Abbreviations

AD: Alzheimer’s Disease
ADNI: Alzheimer’s Disease Neuroimaging Initiative
CER: Columns Exclusion Ratio
DREAMER: Data Readiness for Machine learning Research
FHS: Framingham Heart Study
kNN: k Nearest Neighbors
MAD: Median Absolute Deviation
ML: Machine Learning
MV: Missing Values
PC: Pearson Correlation
RER: Rows Exclusion Ratio
RF: Random Forest
SCC: Shared Computing Cluster
SGD: Stochastic Gradient Descent
WDBC: Wisconsin Diagnosis Breast Cancer

## References

[1] Sarker IH (2021). Machine learning: algorithms, real-world applications and research directions. SN Comput Sci.

[2] Lawrence ND. Data readiness levels. arXiv preprint arXiv:170502245. 2017.

[3] Dakka MA, Nguyen TV, Hall JMM, Diakiw SM, VerMilyea M, Linke R (2021). Automated detection of poor-quality data: case studies in healthcare. Sci Rep.

[4] Austin CC. A path to big data readiness. In: 2018 IEEE International Conference on Big Data (Big Data). IEEE; 2018. pp. 4844–53.

[5] Barham H, Daim T (2020). The use of readiness assessment for big data projects. Sustain Cities Soc.

[6] de Hond AAH, Leeuwenberg AM, Hooft L, Kant IMJ, Nijman SWJ, van Os HJA (2022). Guidelines and quality criteria for artificial intelligence-based prediction models in healthcare: a scoping review. NPJ Digit Med.

[7] Castelijns LA, Maas Y, Vanschoren J. The abc of data: A classifying framework for data readiness. In: Machine Learning and Knowledge Discovery in Databases: International Workshops of ECML PKDD 2019, Würzburg, Germany, September 16–20, 2019, Proceedings, Part I. Springer; 2020. pp. 3–16.

[8] Feurer M, Klein A, Eggensperger K, Springenberg J, Blum M, Hutter F. Efficient and Robust Automated Machine Learning. In Advances in neural information processing systems. 2015;28:2962–2970.

[9] Gebru T, Morgenstern J, Vecchione B, Vaughan JW, Wallach H, Iii HD (2021). Datasheets for datasets. Commun ACM.

[10] Bender EM, Friedman B (2018). Data statements for natural language processing: toward mitigating system bias and enabling better science. Trans Assoc Comput Linguist.

[11] Arnold M, Bellamy RKE, Hind M, Houde S, Mehta S, Mojsilović A (2019). FactSheets: increasing trust in AI services through supplier’s declarations of conformity. IBM J Res Dev.

[12] Holland S, Hosny A, Newman S, Joseph J, Chmielinski K. The dataset nutrition label: A framework to drive higher data quality standards. arXiv preprint arXiv:180503677. Hart Publishing. 2020;12(12):1.

[13] Mitchell M, Wu S, Zaldivar A, Barnes P, Vasserman L, Hutchinson B, et al. Model cards for model reporting. In: Proceedings of the conference on fairness, accountability, and transparency. 2019. pp. 220–9.

[14] Petersen AH, Ekstrøm CT (2019). dataMaid: your assistant for documenting supervised data quality screening in R. J Stat Softw.

[15] Arslan RC (2019). How to automatically document data with the codebook package to facilitate data reuse. Adv Methods Pract Psychol Sci.

[16] Gupta N, Patel H, Afzal S, Panwar N, Mittal RS, Guttula S, et al. Data Quality Toolkit: automatic assessment of data quality and remediation for machine learning datasets. arXiv Preprint arXiv:210805935. 2021.

[17] Afzal S, Rajmohan C, Kesarwani M, Mehta S, Patel H. Data Readiness Report. In: 2021 IEEE International Conference on Smart Data Services (SMDS). IEEE; 2021. pp. 42–51.

[18] Lavin A, Gilligan-Lee CM, Visnjic A, Ganju S, Newman D, Ganguly S (2022). Technology readiness levels for machine learning systems. Nat Commun.

[19] Zhang A, Xing L, Zou J, Wu JC. Shifting machine learning for healthcare from development to deployment and from models to data. Nat Biomed Eng. London: Nature Publishing Group; 2022;6(12):1330–45. 10.1038/s41551-022-00898-y PMC12063568 35788685

[20] ADNI Dataset. http://adni.loni.usc.edu. Accessed 28 May 2024.

[21] FHS Dataset. https://www.framinghamheartstudy.org. Accessed 28 May 2024.

[22] Street WN, Wolberg WH, Mangasarian OL (1993). Nuclear feature extraction for breast tumor diagnosis. Biomedical image processing and biomedical visualization.

[23] Xu X-Y, Huang X-L, Li Z-M, Gao J, Jiao Z-Q, Wang Y (2020). A scalable photonic computer solving the subset sum problem. Sci Adv.

[24] Oh S (2011). A new dataset evaluation method based on category overlap. Comput Biol Med.

