# Supplementary material for: DREAMER: a computational framework to evaluate readiness of datasets for machine learning
Source: BMC Med Inform Decis Mak. 2024 Jun 4;24:152. doi: 10.1186/s12911-024-02544-w (PMC11149315; doi:10.1186/s12911-024-02544-w)
Supplement: Supplementary file 1 — Supplementary Material 1. [file 12911_2024_2544_MOESM1_ESM.docx]

**Supplementary Notes**

**Supplementary Notes 1** Overview of key data quality measures.

**Class Overlap:** This measure assesses regions where different classes overlap, which can lead to misclassification by machine learning (ML) algorithms. The overlap detection metric examines the dataset to identify data points that are situated in these ambiguous regions, where class boundaries are not well-defined.

**Label Purity:** This metric evaluates the extent of label noise within the dataset. High levels of noisy labels can reduce learning accuracy and increase the number of samples needed for effective model training. The label purity metric calculates the noise ratio and identifies the count of samples with incorrect or misleading labels.

**Class Parity:** Imbalances in class distribution can bias ML models towards the majority class, affecting their performance. The class parity measure helps in analyzing dataset balance by examining parameters like the imbalance ratio and dataset size. It also provides recommended re-sampling strategies to address class imbalance, promoting fairer outcomes during model training.

**Feature Relevance:** This metric assists data scientists in assessing the significance of each feature relative to the target variable and other features within the dataset. Ideally, a feature with high relevance demonstrates a strong correlation with the target variable while maintaining low correlation with other features. This metric is particularly valuable in high-dimensional datasets, where a large number of features may contain redundant or irrelevant information. During the remediation process, features with lower relevance are discarded, ensuring that only those features contributing to the optimal performance of classification tasks are retained.

**Data Homogeneity:** Consistent data formats are essential for building machine learning models and conducting business analytics. This metric allows data scientists to identify format inconsistencies in non-numerical columns and correct them by transforming the data into the desired format.

**Data Fairness:** FAIR data adhere to the principles of *Findability*, *Accessibility*, *Interoperability*, and *Reusability*. This metric detects bias in the dataset, providing a disparate impact score. Data fairness remediation enhances the data quality by reducing bias and improving group fairness through edits to feature values.

**Correlation Detection:** High correlations among attributes in a dataset can lead to multi-collinearity, potentially causing machine learning models to overfit the training data, reducing their performance on unseen test sets. This metric identifies highly correlated features, allowing remediation by removing the features with the highest correlation to others.

**Data Completeness:** This metric identifies missing values and offers imputation mechanisms to improve data modeling. It uses constraint-based and association-based approaches to fill in gaps in the data, enhancing its reliability.

**Outlier Detection:** Outliers are observations that deviate significantly from other data points, indicating they might have been generated by different mechanisms. Outliers can lead to increased misclassification and reduced model performance. The corresponding remediation involves detecting and removing outliers from the dataset.

**Data Duplicates:** Duplicate records not only waste memory and computational resources but also contribute to data imbalance. This metric quantifies the extent of duplication, providing mechanisms for removing redundant records to ensure a cleaner and more efficient dataset.

**Supplementary Notes 2** Overview of the common data readiness tools.

*AutoML* frameworks operate on structured datasets and are designed to automate feature engineering and model building through advanced Bayesian optimization methods. However, these frameworks do not inherently assess the quality of the input data.

*Datasheets* for Datasets aim to enhance transparency and accountability in machine learning by providing structured documentation about datasets. This approach helps researchers select datasets that align with their specific tasks, addressing critical, ethical, and technical considerations.

*Data Statements* are conceptually similar to Datasheets but focus on linguistic datasets, particularly those used in Natural Language Processing (NLP) applications. They aim to provide comprehensive documentation for NLP datasets, including information about the context, collection methods, and ethical considerations.

*FactSheets* are intended to build trust in AI services by providing detailed documentation covering aspects such as purpose, performance, safety, and security. By presenting standardized information on various attributes—such as data fairness, data lineage, training data, trained model, and application domain—FactSheets aim to bridge the knowledge gap between AI service producers and consumers.

*Dataset Nutrition* Label offers a detailed breakdown of a dataset's key components, presented in a standardized format. It encompasses a variety of qualitative and quantitative modules, derived from multiple statistical and probabilistic models. This Label aids ML researchers in selecting the most appropriate dataset for their needs, thus enhancing the overall quality of AI systems by utilizing more robust training data for modeling.

*Model Cards* serve as a framework for constructing transparent model reports, providing comprehensive documentation of the performance characteristics of ML models. A typical Model Card includes information such as model details (version, type, training algorithm, etc.), intended use cases, performance metrics (e.g., accuracy, decision thresholds), evaluation data (datasets, preprocessing steps), training data, and other quantitative analyses. By offering such extensive documentation, Model Cards facilitate better understanding and evaluation of ML models.

*Datamaid* offers automated tools for data quality assessment in R, allowing users to identify potential issues within dataset variables. The package auto-generates a human-readable report that summarizes the results of data quality screening. While Datamaid does not include functionalities for correcting errors in the data, it facilitates interactive exploration of datasets, enabling users to identify potential sources of errors and address them manually.

*Codebook* is another R package that provides comprehensive documentation for a given dataset. It generates human- and machine-readable metadata, encompassing key information such as data statistics, data distribution, and details on missing values. This structured documentation helps researchers better understand their datasets and guides further data processing and analysis.

The *IBM Data Quality Toolkit* is a comprehensive library of key quality metrics, along with associated remediation methods designed to improve the readiness of structured datasets for machine learning tasks. The toolkit comprises three primary components: *Data Quality Measurement*, *Data Remediation*, and *Data Readiness Reporting*. Data Quality Measurement involves assessing the quality of the data at the initial stages of the data science lifecycle, identifying factors that contribute to low-quality data, and providing recommendations for corrective actions. The Data Remediation component includes specific techniques to address the identified issues, thereby improving the dataset's suitability for machine learning applications. The Data Readiness Report is a sharable document that encapsulates the baseline quality metrics of the dataset, along with a detailed record of all operations and remediations applied to it. This comprehensive report serves as a valuable resource for maintaining quality calibration throughout the data science workflow, ensuring that the integrity and readiness of the data are properly documented and communicated.

The *Data Readiness Report* centers on data quality by subjecting the data to a series of cleaning, validation, and transformation analyses before it is deemed suitable for machine learning tasks. This report serves as supplementary documentation for a dataset, providing data scientists with detailed insights into the dataset's quality. The report identifies and documents data properties based on various quality criteria, contributing to enhanced transparency and explainability. It also encompasses a record of all data assessment operations and applied transformations, offering a complete overview of the dataset's readiness for machine learning applications. The report expresses data properties using AI-relevant terminology, making it easier for AI practitioners to understand the dataset's characteristics. Additionally, the Data Readiness Report serves as a repository for tracking data operations, detailing who has modified or transformed the data, how, and at what stage. This feature facilitates user interactions with the data, reducing the need for repetitive data exploration and analysis, ultimately expediting the development of machine learning models. The Data Readiness Report serves as a comprehensive framework for assessing data quality, providing valuable insights for data practitioners. This framework encompasses several key components:

*Basic Metadata:* Contains essential details about the dataset, such as its name, version, generation date, and type of data (e.g., structured, unstructured, or time series).

*Summary of Quality and Readiness Assessment:* A high-level overview of the quality and readiness evaluation.

*Baseline Data Profile:* Offers descriptive statistics of the original dataset, including the number of rows and columns, data types, maximum and minimum values for each column, distribution of data, and information on missing data.

*Baseline Quality and Readiness Assessment:* Evaluates the dataset against various quality metrics, providing detailed explanations and recommended remedial actions to address any identified issues.

*Updated Data Profile:* Describes the dataset's characteristics after applying remediation strategies, highlighting any changes.

*Updated Quality and Readiness Assessment:* Reflects the updated evaluation of data quality following remediation.

*Lineage of Operation:* Tracks the source of data operations, identifying the individuals or systems involved in various modifications or transformations.

*Data Governance:* Outlines governance policies applied to the dataset.

*References:* Provides detailed explanations of the metrics used and the remediation processes applied.

These components collectively offer a robust framework for assessing data quality and readiness for machine learning applications, while also ensuring data transparency, traceability, and governance.
