# Supplementary material for: DREAMER: a computational framework to evaluate readiness of datasets for machine learning
Source: BMC Med Inform Decis Mak. 2024 Jun 4;24:152. doi: 10.1186/s12911-024-02544-w (PMC11149315; doi:10.1186/s12911-024-02544-w)
Supplement: Supplementary file 2 — Supplementary Material 2. [file 12911_2024_2544_MOESM2_ESM.docx]

**Supplementary Figures**

**Supplementary Fig. 1** Distribution of class labels in the FHS, ADNI, and WDBC datasets. (a) Pie chart depicting the distribution of class labels in the FHS dataset, with two categories: "No dementia" and "Probable dementia." (b) Pie chart illustrating the distribution of class labels in the ADNI dataset, with five categories: "AD," "CN," "EMCI," "LMCI," and "SMC." (c) Pie chart showing the distribution of class labels in the WDBC dataset, with two categories: "Benign" (non-cancerous) and "Malignant" (cancerous).

**a**


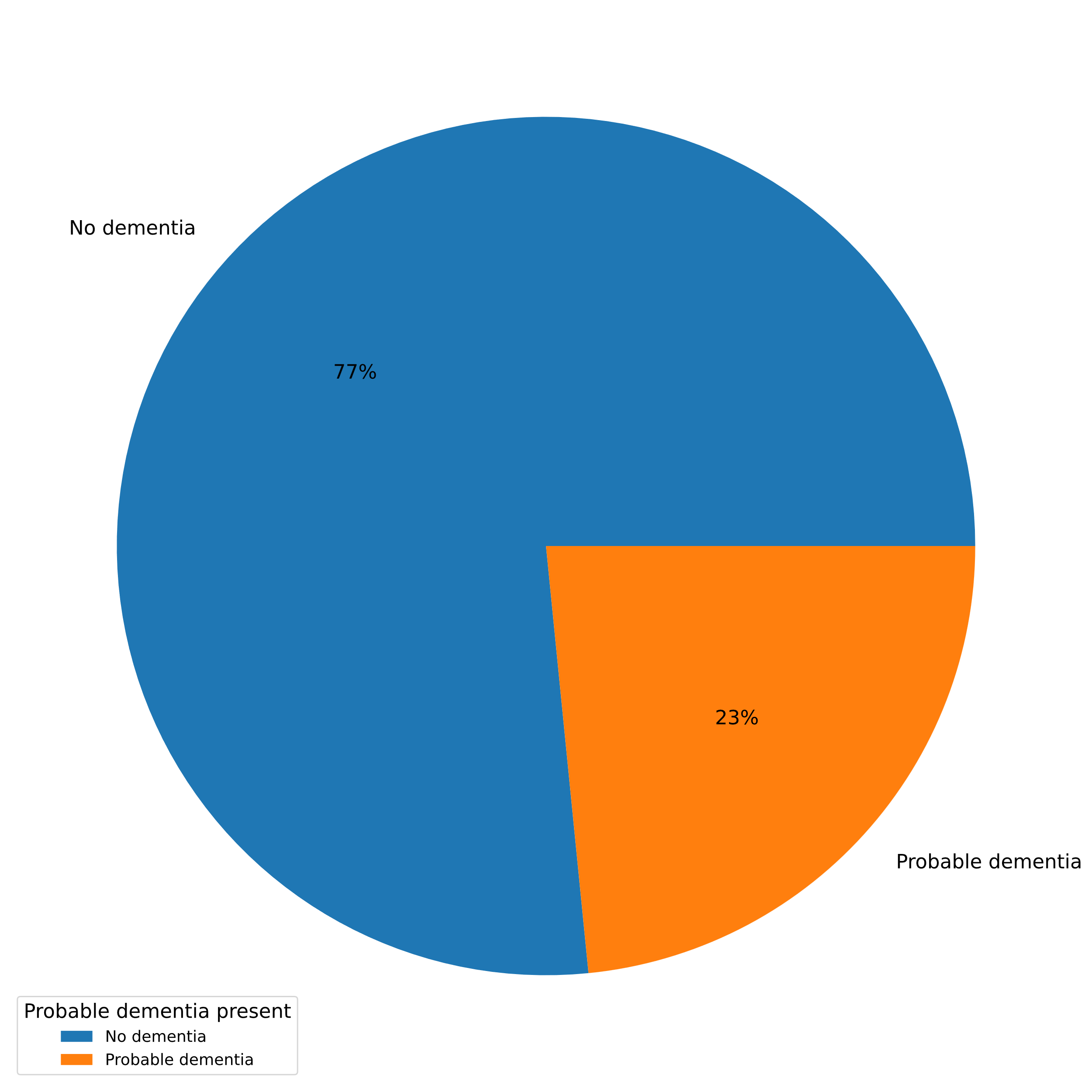

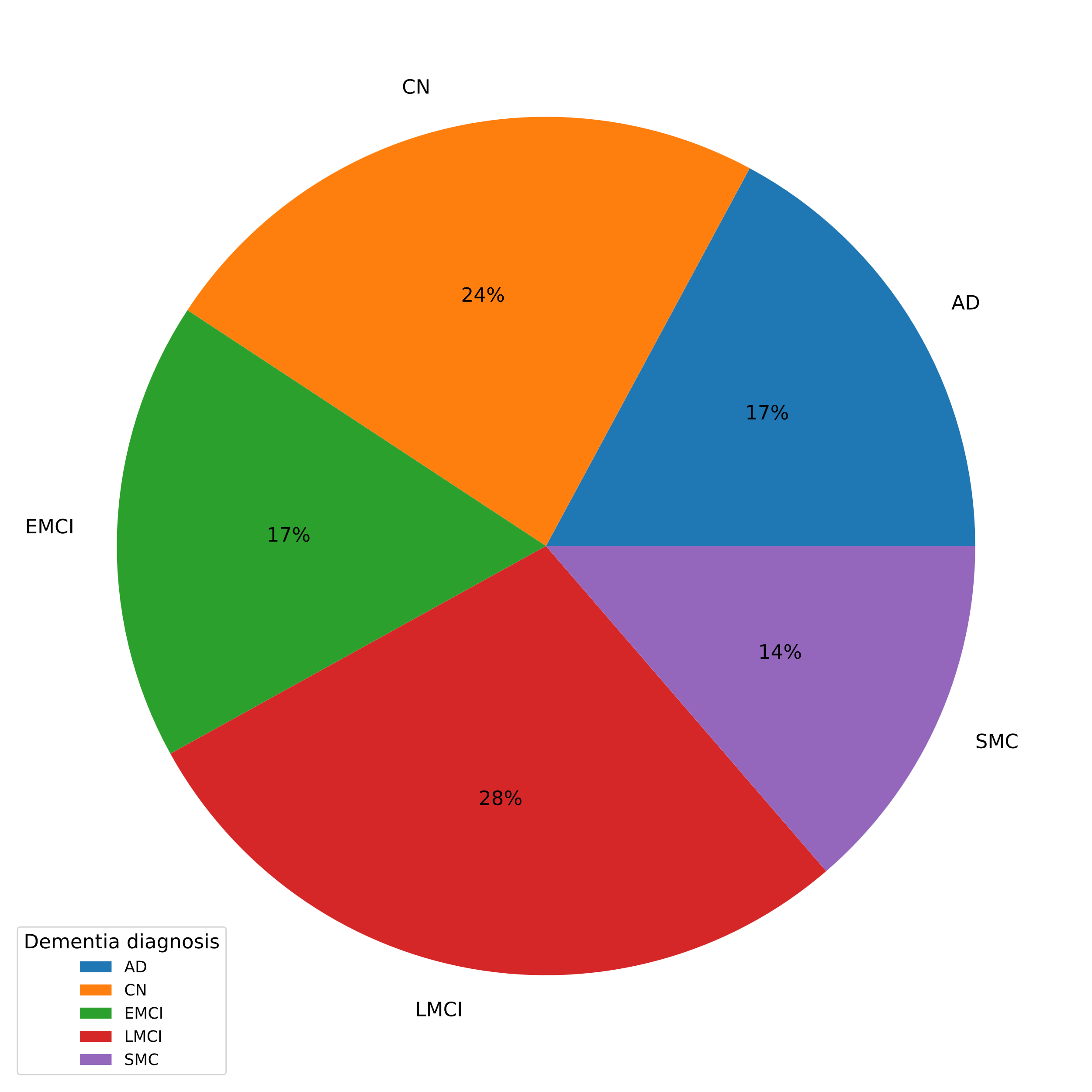


**b**

**
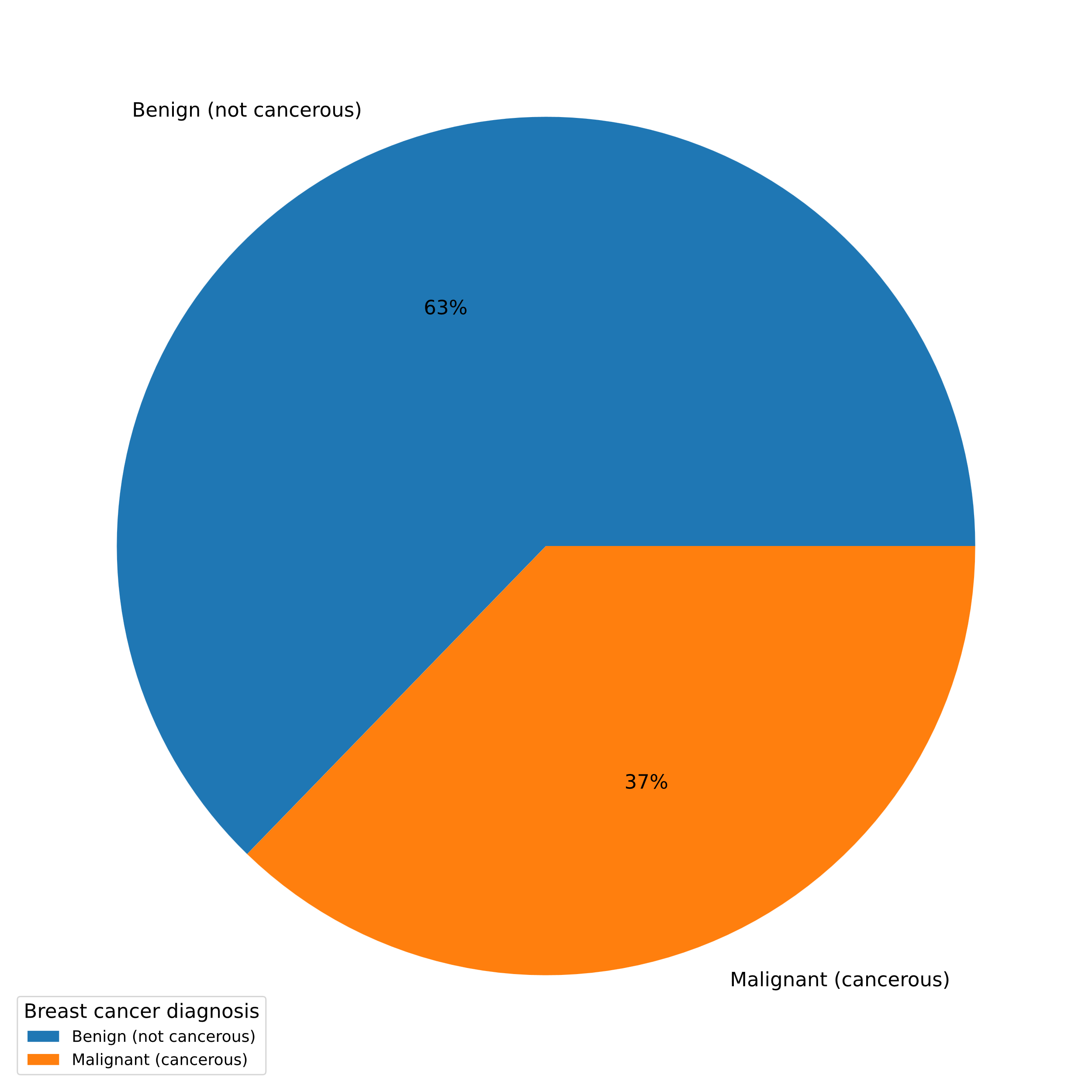
**

**c**

**Supplementary Fig. 2** Heatmap analysis of correlations between weights and scores of data quality measures across ten different runs of the DREAMER algorithm on the FHS, ADNI, and WDBC datasets.

(**a**) Heatmap outlining the correlation between data quality scores and both classification and clustering accuracy in different runs of DREAMER on the FHS dataset.

**
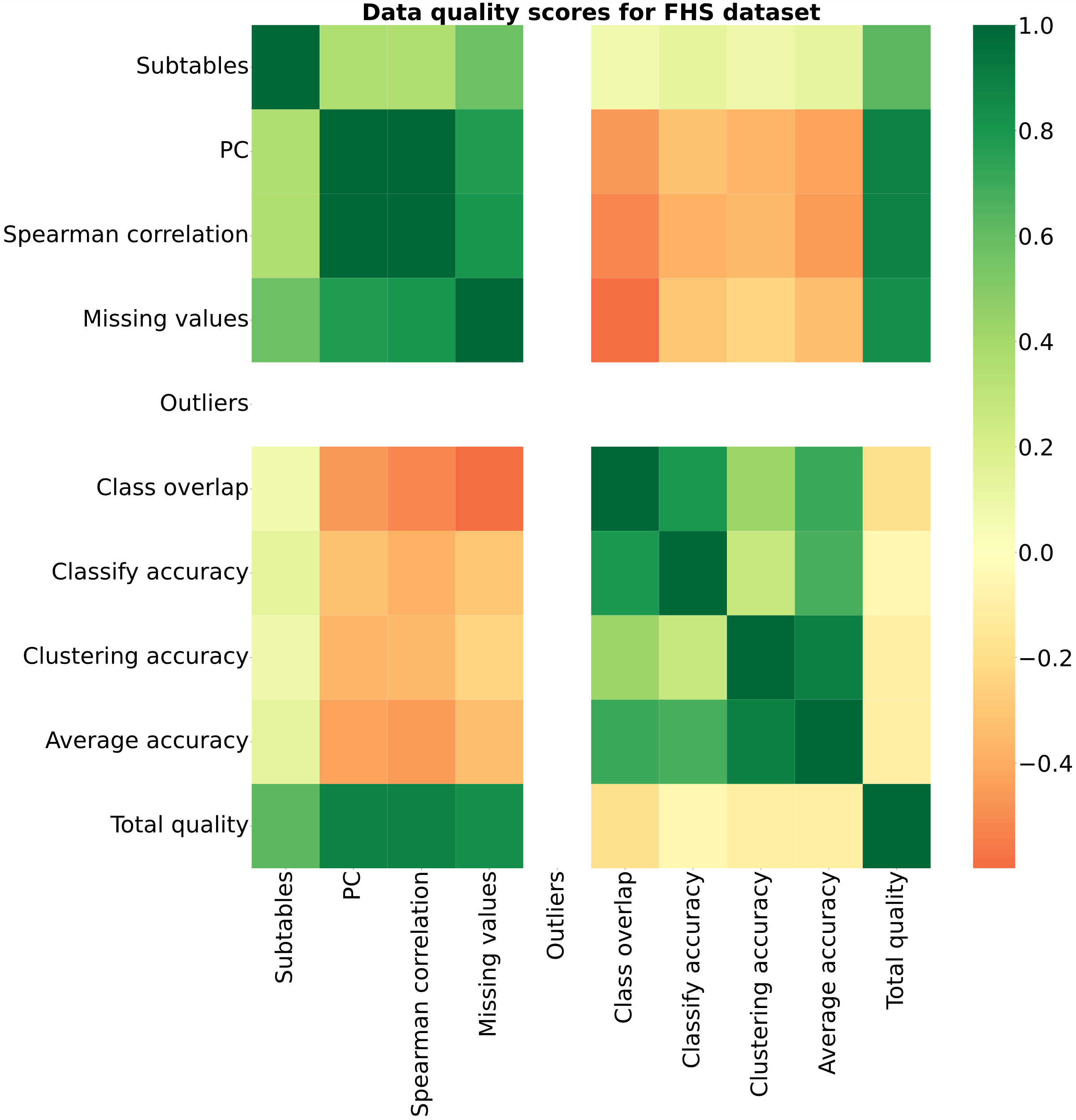
**

(**b**) Heatmap illustrating the correlation between data quality weights and both classification and clustering accuracy in different runs of DREAMER on the FHS dataset.

**
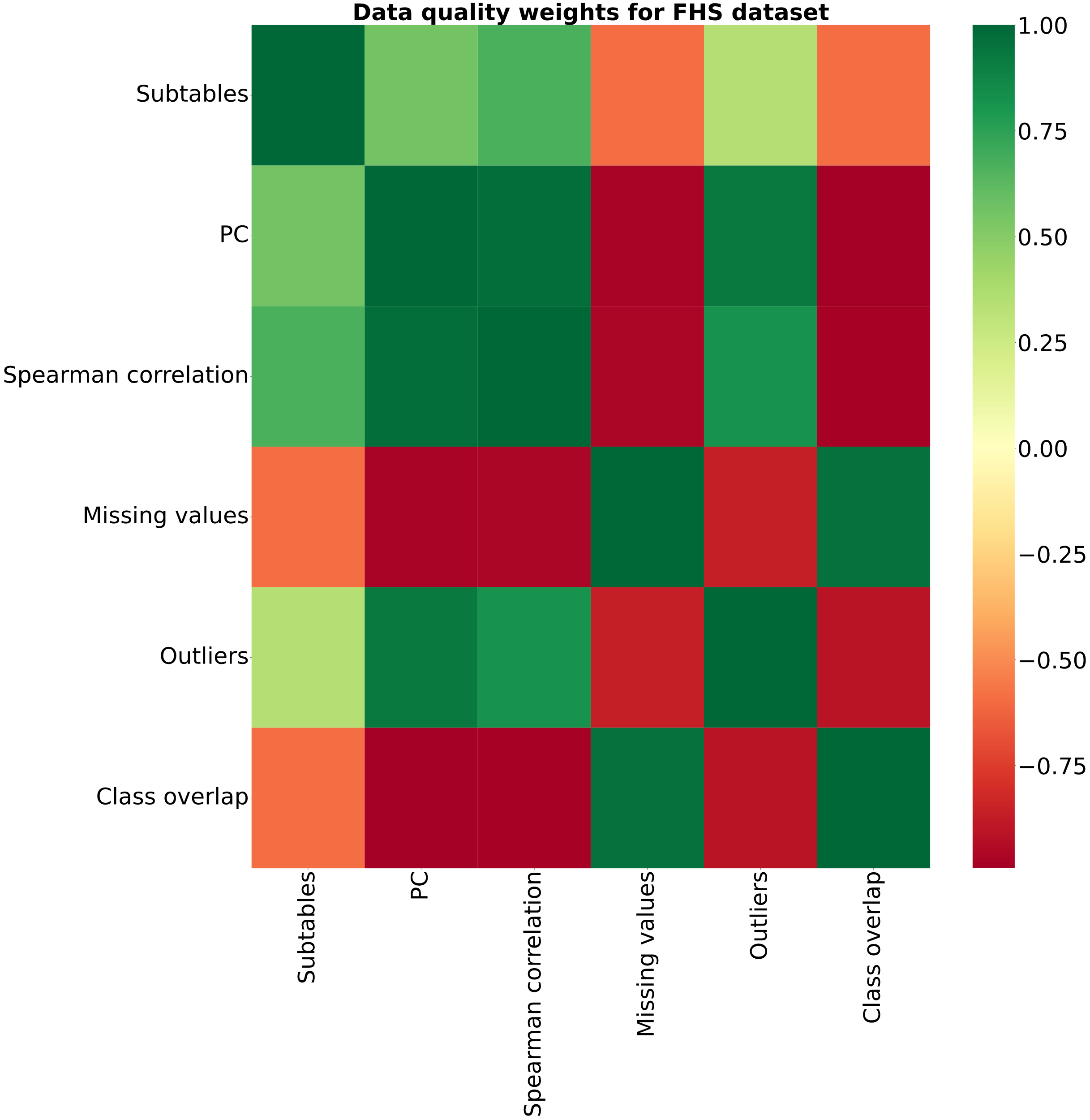
**

(**c**) Heatmap outlining the correlation between data quality scores and both classification and clustering accuracy in different runs of DREAMER on the ADNI dataset.


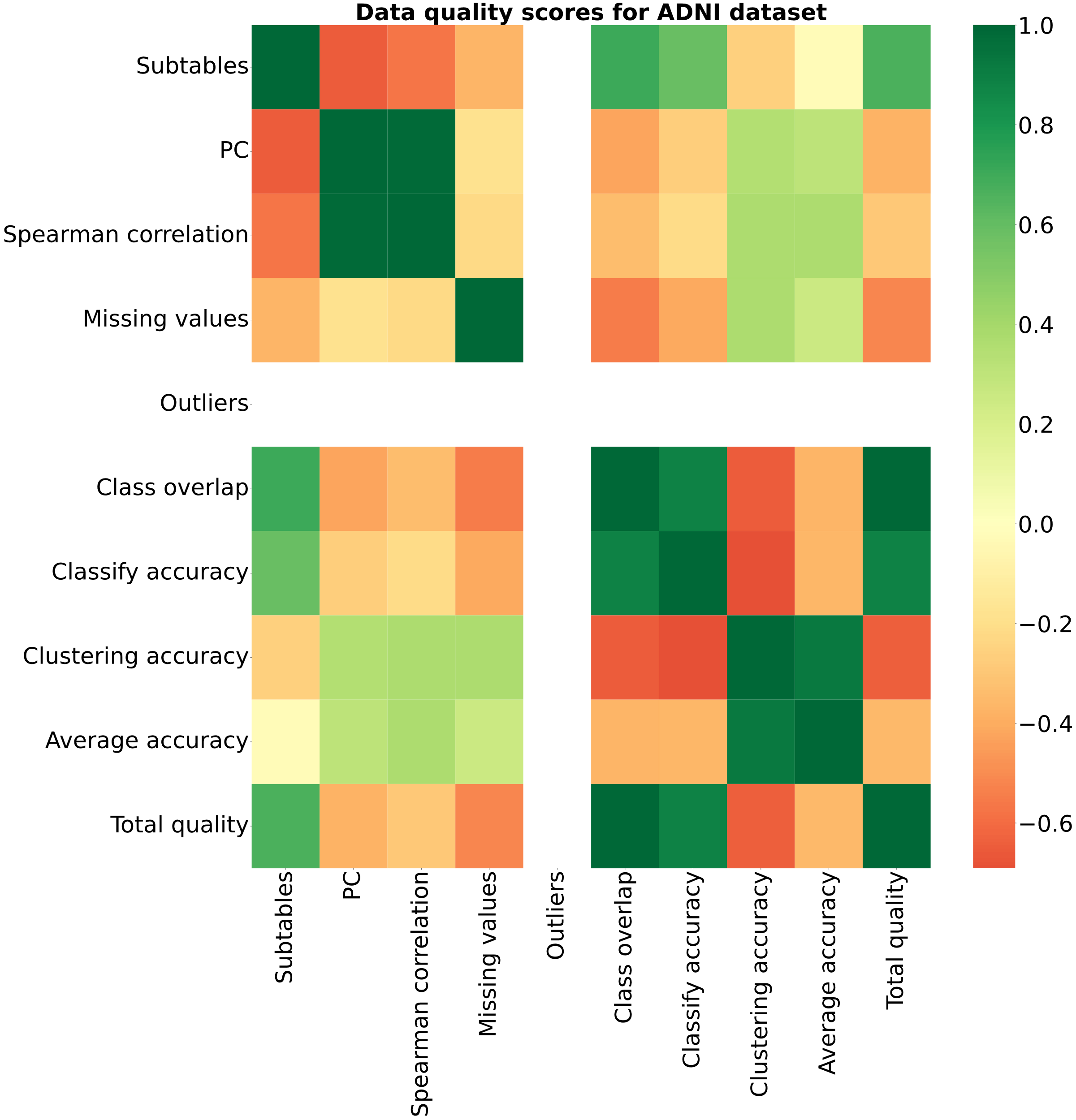


(**d**) Heatmap illustrating the correlation between data quality weights and both classification and clustering accuracy in different runs of DREAMER on the ADNI dataset.


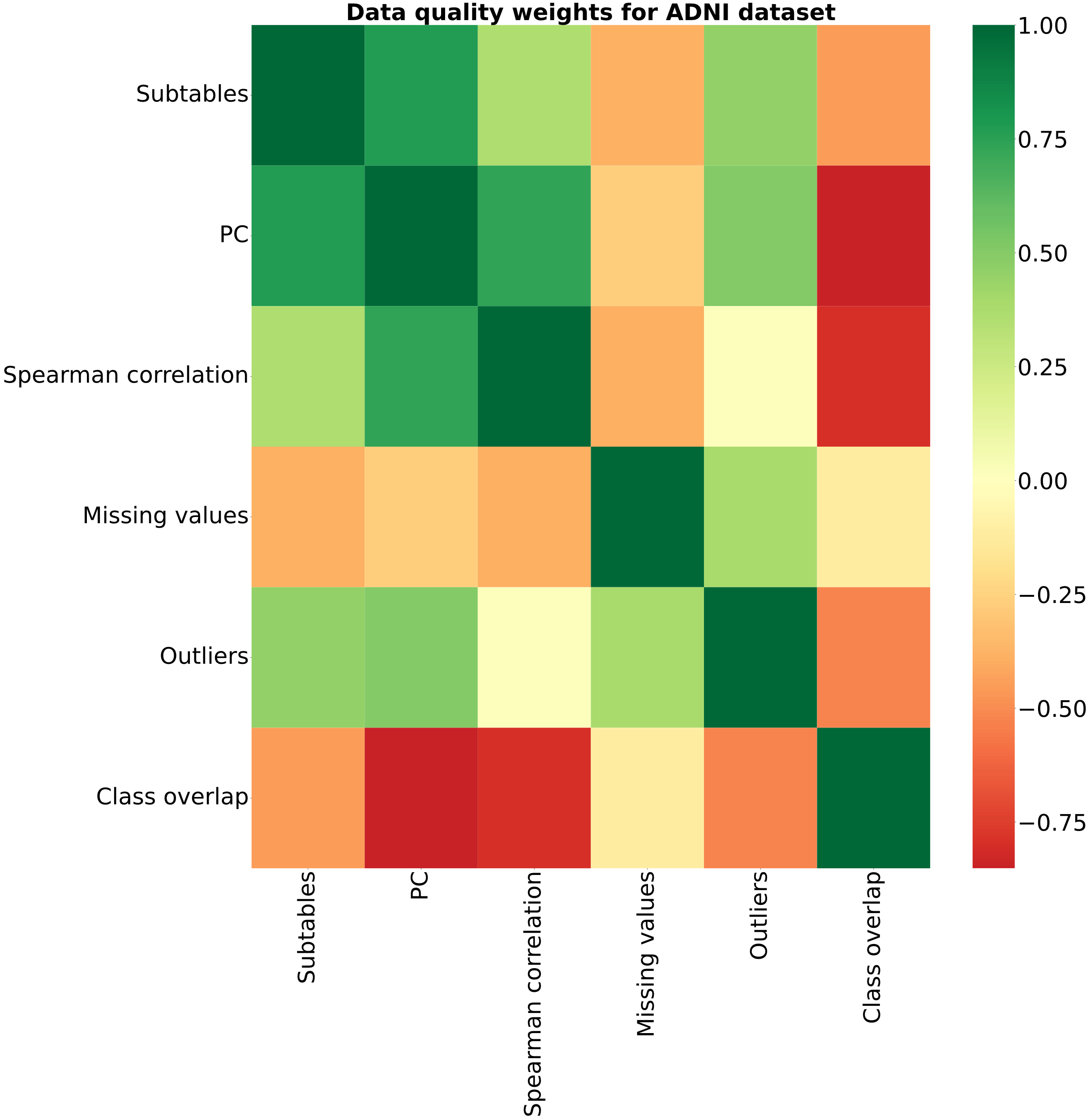


(**e**) Heatmap outlining the correlation between data quality scores and both classification and clustering accuracy in different runs of DREAMER on the WDBC dataset.


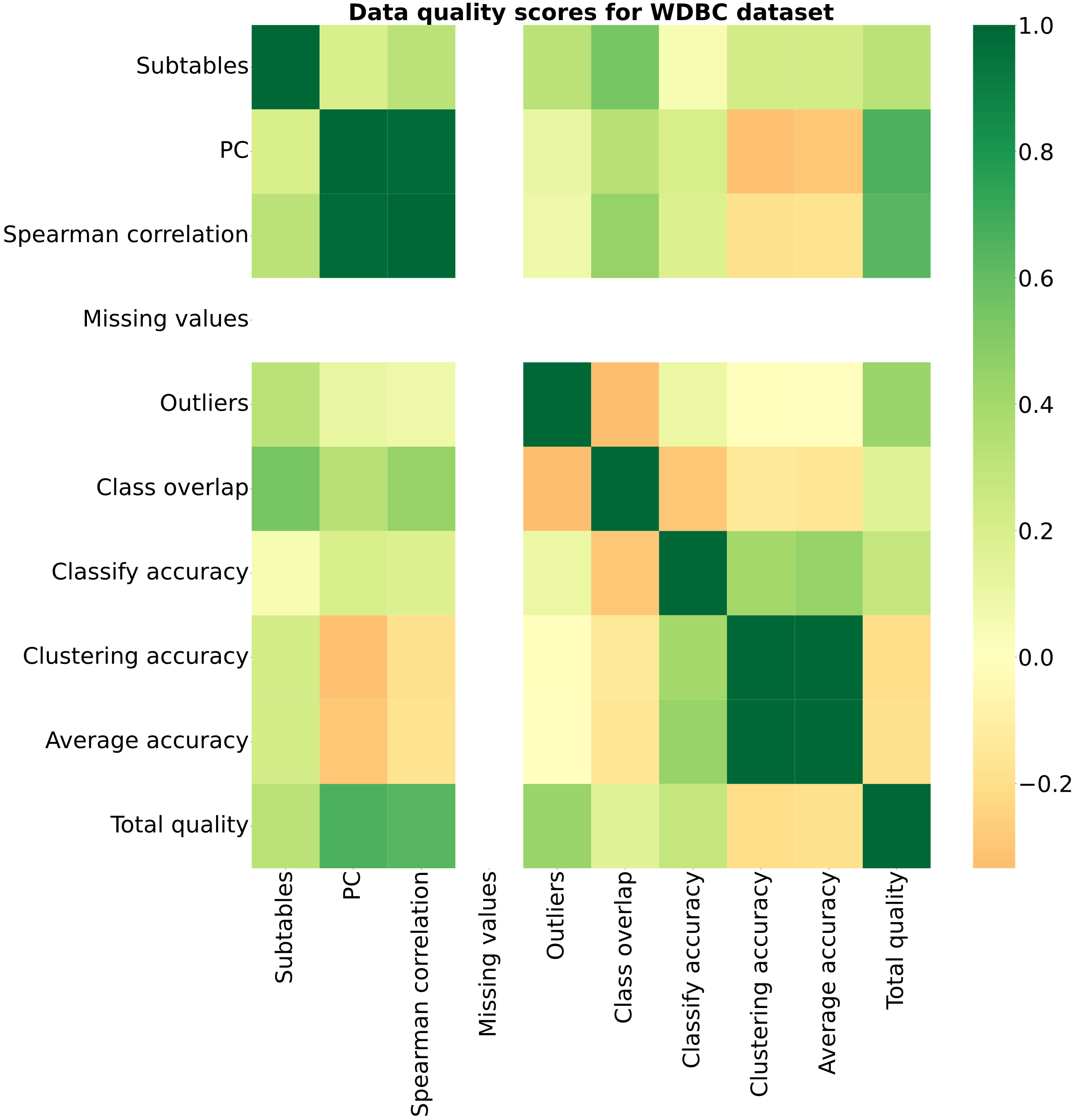


(**f**) Heatmap illustrating the correlation between data quality weights and both classification and clustering accuracy in different runs of DREAMER on the WDBC dataset.


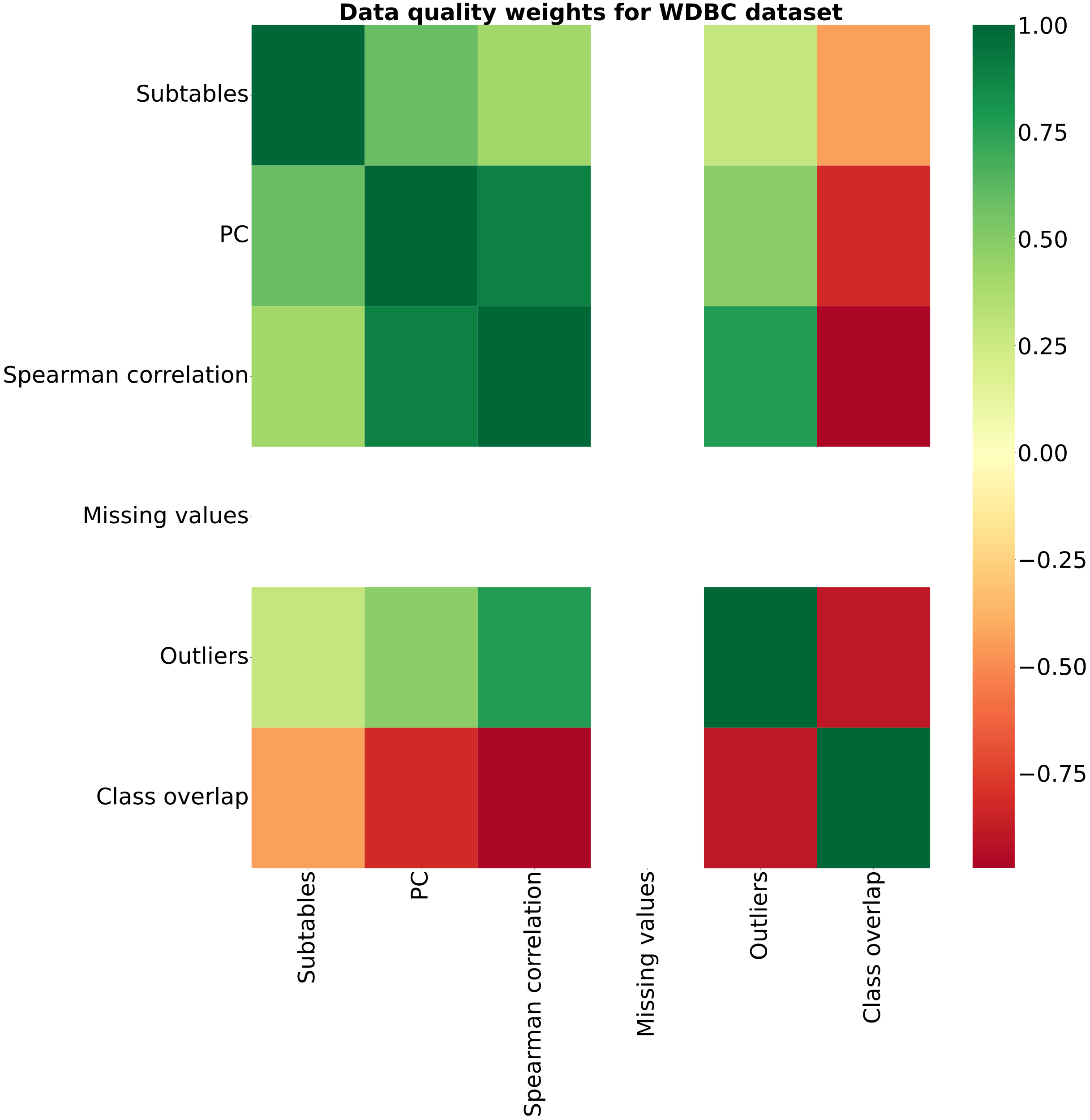


**Supplementary Fig. 3** Regression analysis exploring the correlation between weights and scores of data quality measures across ten different runs of the DREAMER algorithm on the FHS, ADNI, and WDBC datasets. The blue dots represent the best sub-table from each run, and the blue lines indicate the estimated regression function. The density diagrams along the main diagonal represent the Probability Density Function (PDF) for each feature, estimated using the Kernel Density Estimation (KDE) method.

(**a**) Correlogram illustrating the relationship between data quality scores and both classification and clustering accuracy in different runs of DREAMER on the FHS dataset, derived through regression and KDE methods.


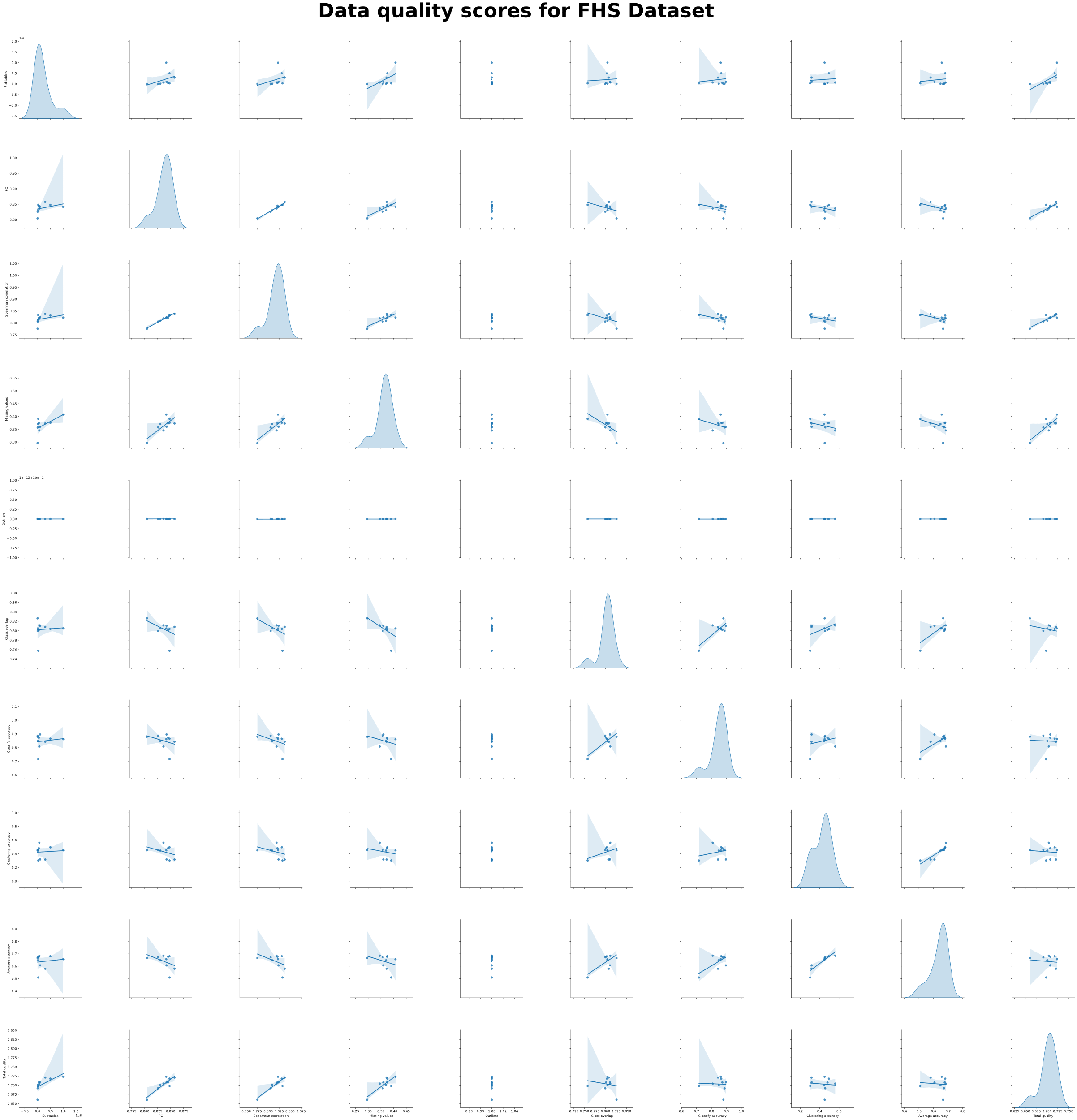


(**b**) Correlogram depicting the relationship between data quality weights across different runs of DREAMER on the FHS dataset, using both regression and KDE methods.


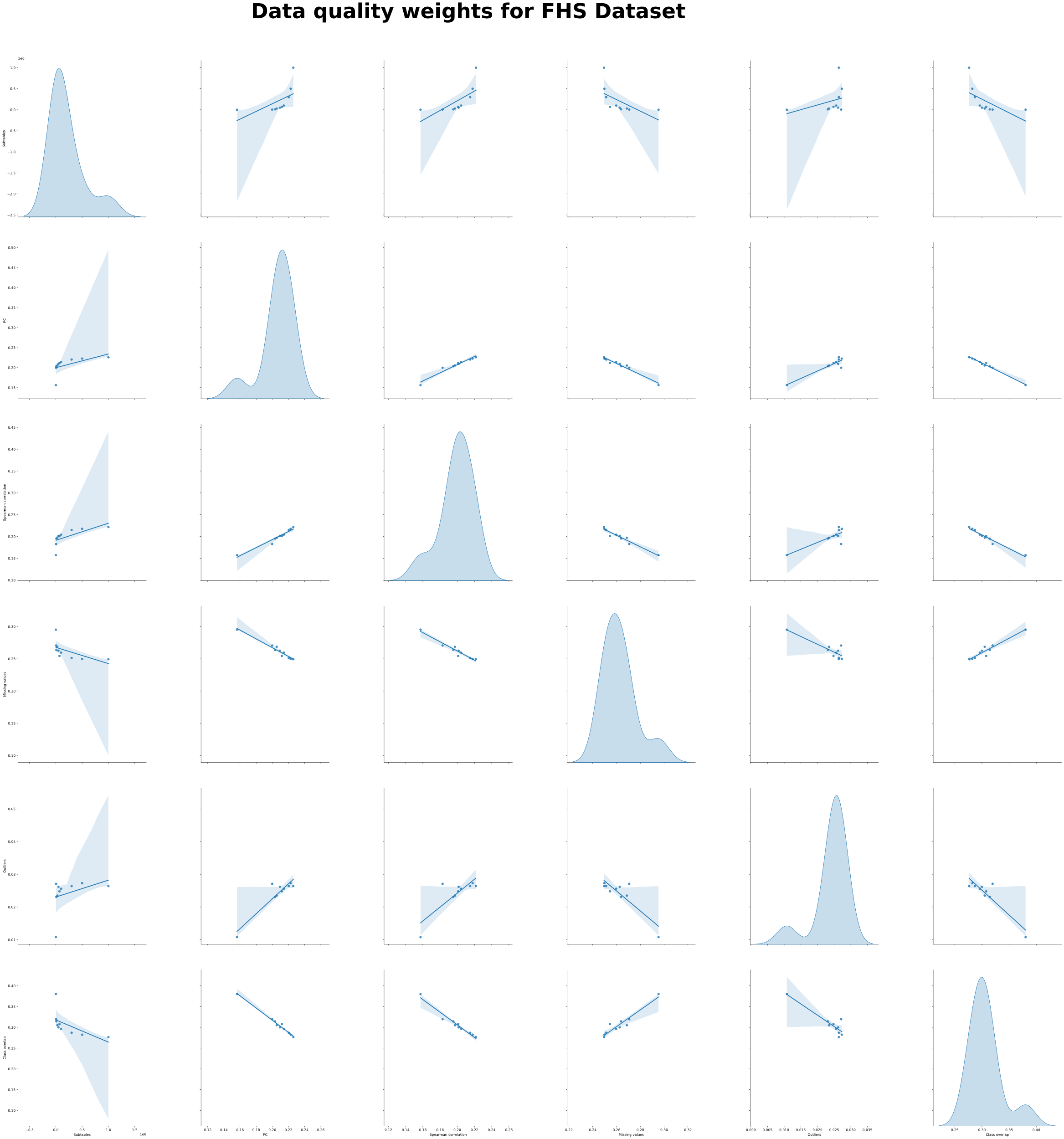


­­­­(**c**) Correlogram illustrating the relationship between data quality scores and both classification and clustering accuracy in different runs of DREAMER on the ADNI dataset, derived through regression and KDE methods.


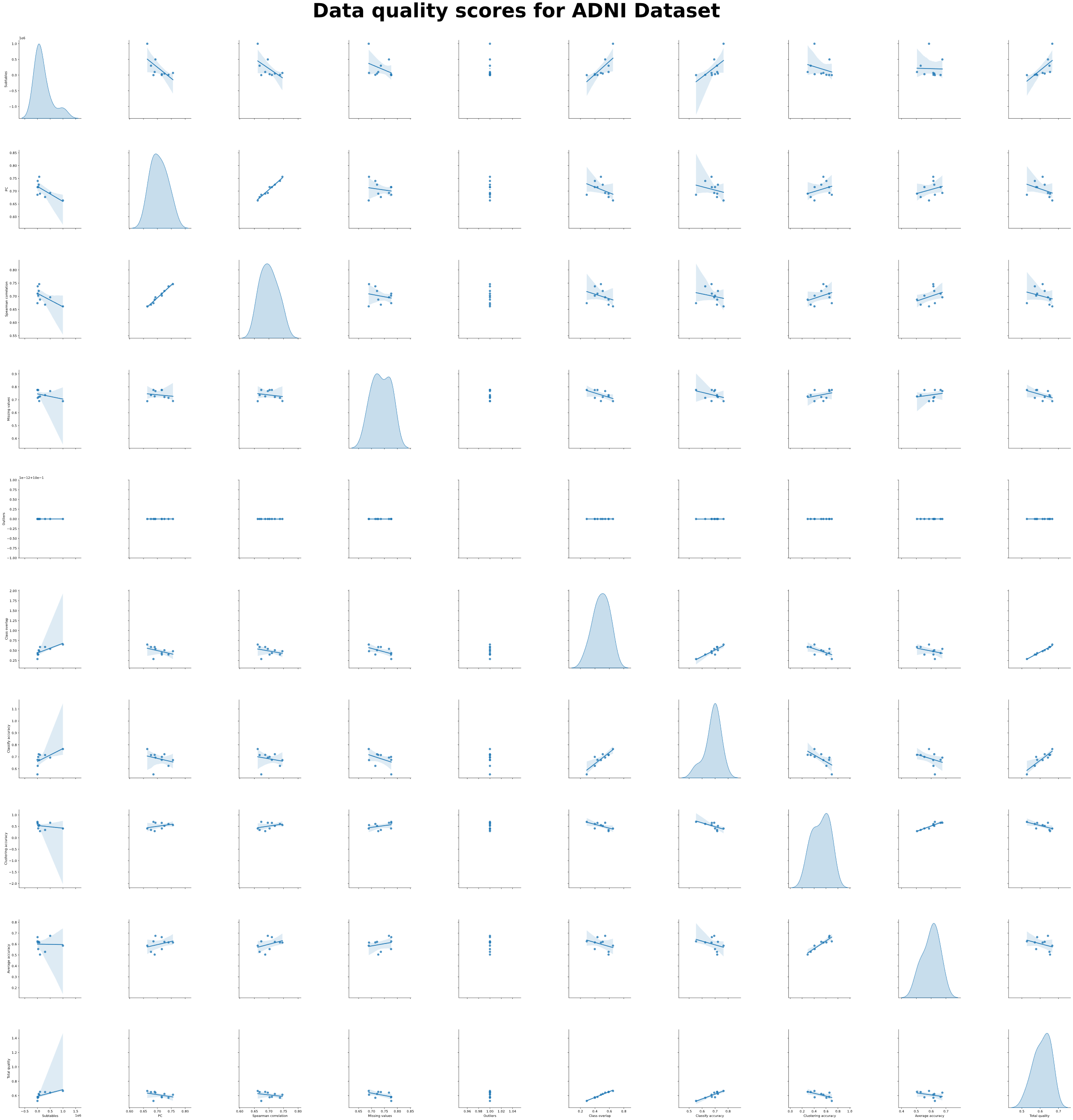


(**d**) Correlogram depicting the relationship between data quality weights across different runs of DREAMER on the ADNI dataset, using both regression and KDE methods.


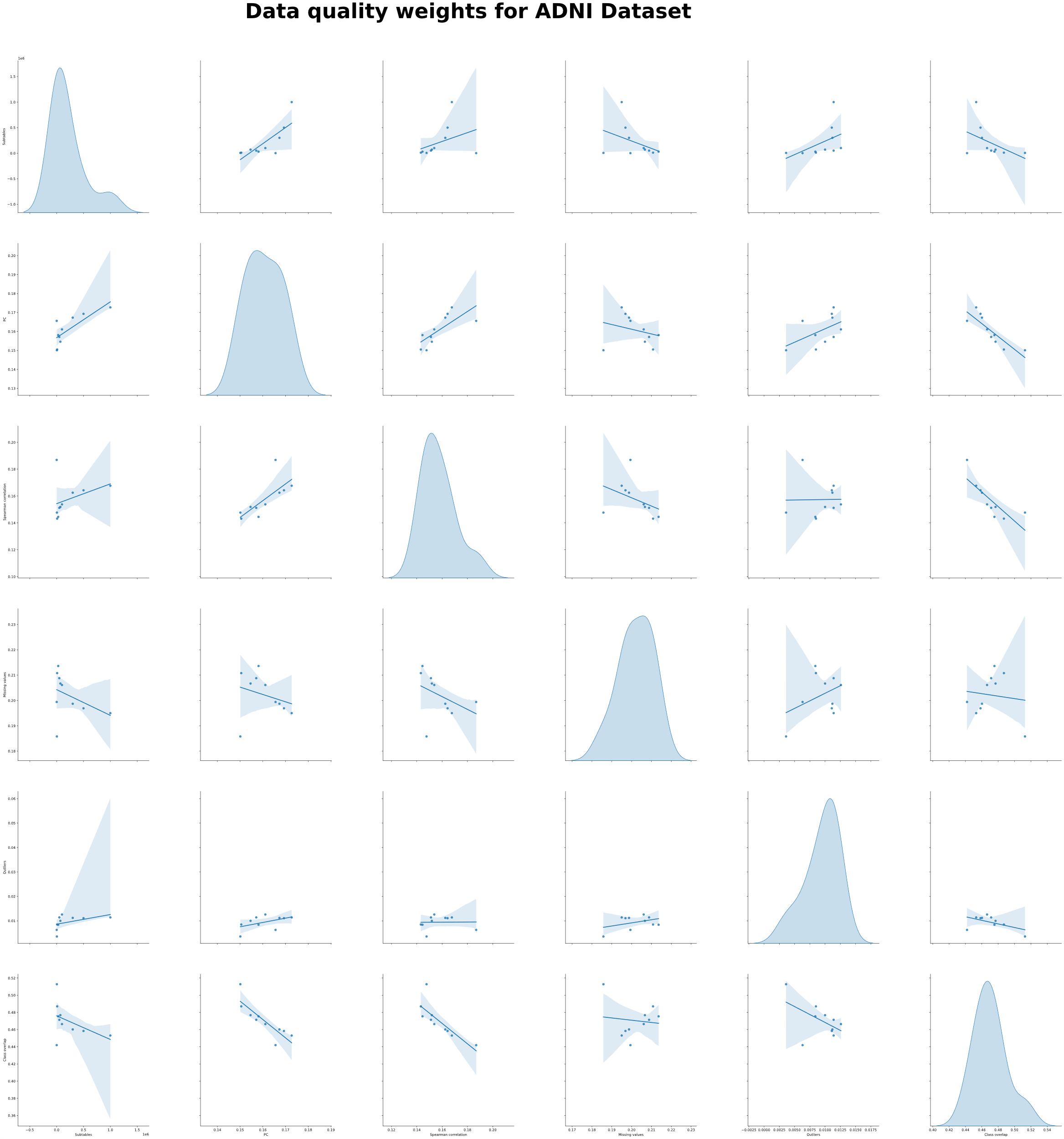


**­­­**(**e**) Correlogram illustrating the relationship between data quality scores and both classification and clustering accuracy in different runs of DREAMER on the WDBC dataset, derived through regression and KDE methods.

**
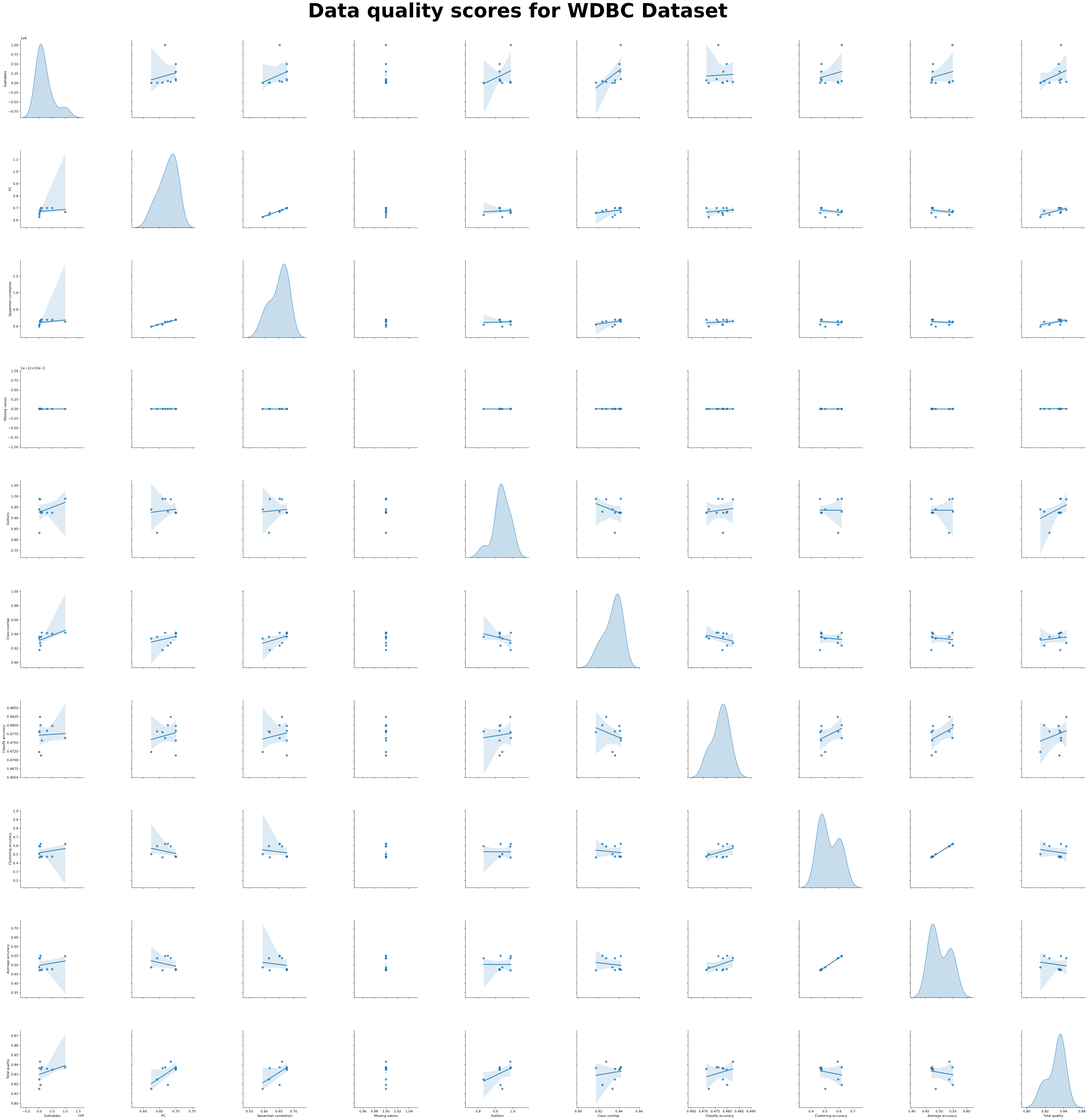
**

(**f**) Correlogram depicting the relationship between data quality weights across different runs of DREAMER on the WDBC dataset, using both regression and KDE methods.

**
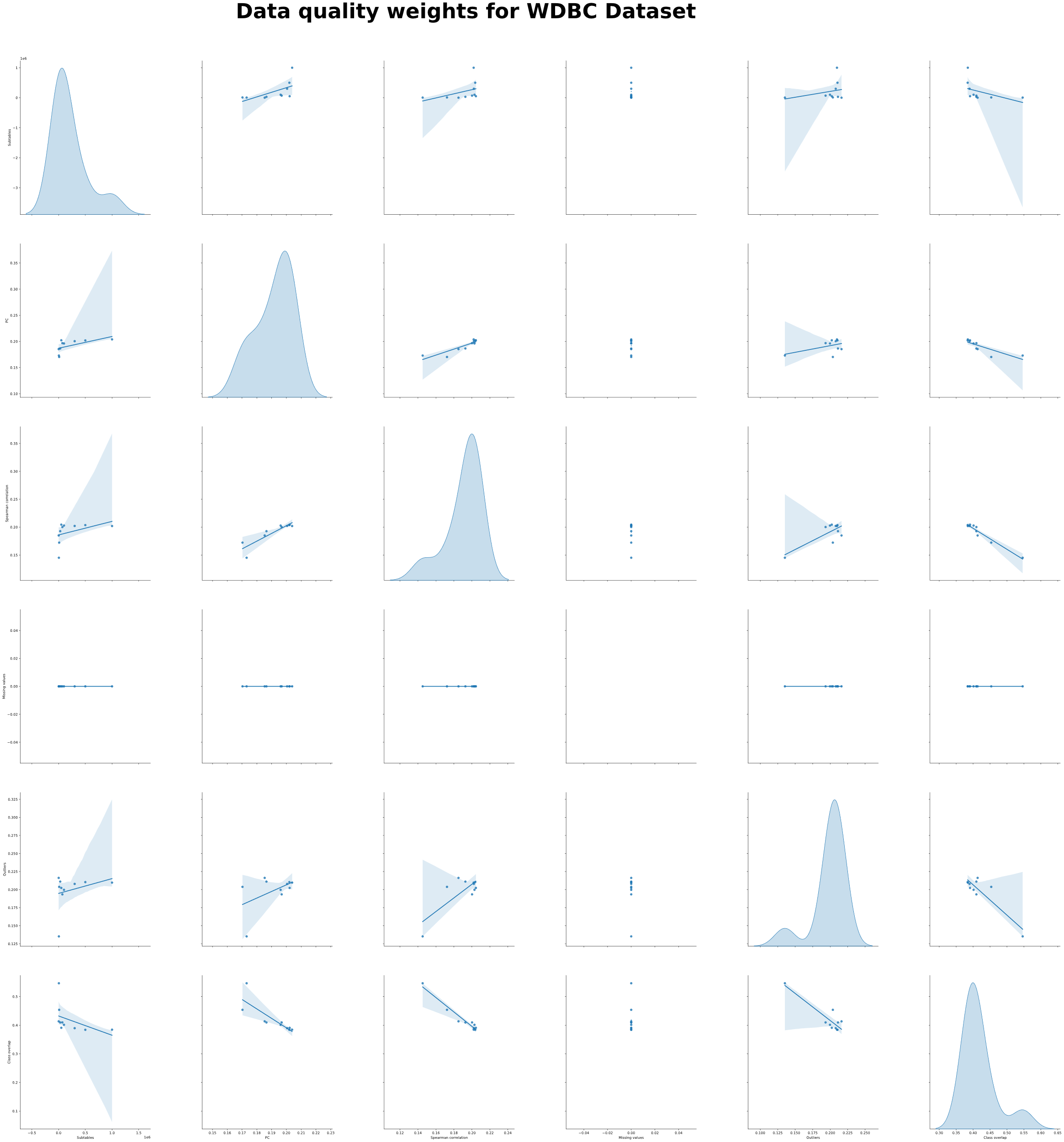
**
