# Supplementary material for: DREAMER: a computational framework to evaluate readiness of datasets for machine learning
Source: BMC Med Inform Decis Mak. 2024 Jun 4;24:152. doi: 10.1186/s12911-024-02544-w (PMC11149315; doi:10.1186/s12911-024-02544-w)
Supplement: Supplementary file 3 — Supplementary Material 3. [file 12911_2024_2544_MOESM3_ESM.docx]

**Supplementary Tables**

**Supplementary Table 1** Mean ranges of data quality features with 95% confidence intervals, calculated under the assumption of a normal distribution.

(**a**) FHS dataset

| Run | Sub-tables | PC | Spearman correlation | Missing values | Outliers | Class overlap | Classify accuracy | Clustering accuracy | Total quality |
| --- | --- | --- | --- | --- | --- | --- | --- | --- | --- |
| 1 | 1000 | (0.7655, 0.7676) | (0.7341, 0.7364) | (0.266, 0.2686) | (0.9572, 0.9764) | (0.7991, 0.8011) | (0.8411, 0.8469) | (0.4444, 0.453) | (0.6284, 0.6298) |
| 2 | 5000 | (0.7684, 0.7695) | (0.7372, 0.7384) | (0.268, 0.2693) | (0.9193, 0.932) | (0.7999, 0.8008) | (0.8466, 0.8489) | (0.4348, 0.4383) | (0.6418, 0.6425) |
| 3 | 10000 | (0.7677, 0.7685) | (0.7367, 0.7376) | (0.2678, 0.2687) | (0.9339, 0.9423) | (0.7999, 0.8005) | (0.8448, 0.8465) | (0.439, 0.4414) | (0.644, 0.6445) |
| 4 | 30000 | (0.7673, 0.7677) | (0.7361, 0.7366) | (0.2685, 0.269) | (0.9347, 0.9395) | (0.8, 0.8004) | (0.8458, 0.8469) | (0.4361, 0.4376) | (0.6411, 0.6415) |
| 5 | 50000 | (0.7676, 0.7679) | (0.7364, 0.7368) | (0.2687, 0.2691) | (0.9306, 0.9345) | (0.8002, 0.8004) | (0.8464, 0.8472) | (0.4354, 0.4365) | (0.6443, 0.6445) |
| 6 | 70000 | (0.7673, 0.7676) | (0.7362, 0.7365) | (0.2688, 0.2692) | (0.9321, 0.9354) | (0.8003, 0.8006) | (0.8469, 0.8476) | (0.4363, 0.4373) | (0.6485, 0.6487) |
| 7 | 100000 | (0.7673, 0.7675) | (0.7362, 0.7364) | (0.2687, 0.269) | (0.9324, 0.9351) | (0.8002, 0.8004) | (0.8464, 0.847) | (0.4356, 0.4364) | (0.6455, 0.6457) |
| 8 | 300000 | (0.7674, 0.7675) | (0.7362, 0.7364) | (0.2688, 0.2689) | (0.9302, 0.9318) | (0.8002, 0.8003) | (0.8468, 0.8472) | (0.4357, 0.4362) | (0.6491, 0.6492) |
| 9 | 500000 | (0.7674, 0.7675) | (0.7362, 0.7364) | (0.2687, 0.2689) | (0.9299, 0.9311) | (0.8003, 0.8004) | (0.8466, 0.8468) | (0.4357, 0.4361) | (0.6498, 0.6499) |
| 10 | 1000000 | (0.7674, 0.7674) | (0.7362, 0.7363) | (0.2688, 0.2689) | (0.9308, 0.9316) | (0.8004, 0.8004) | (0.8467, 0.8469) | (0.4356, 0.4359) | (0.6496, 0.6497) |

(**b**) ADNI dataset

| Run | Sub-tables | PC | Spearman correlation | Missing values | Outliers | Class overlap | Classify accuracy | Clustering accuracy | Total quality |
| --- | --- | --- | --- | --- | --- | --- | --- | --- | --- |
| 1 | 1000 | (0.7451, 0.7473) | (0.7297, 0.7318) | (0.7505, 0.7532) | (0.9410, 0.9636) | (0.1390, 0.1423) | (0.5071, 0.5105) | (0.6152, 0.6218) | (0.4773, 0.4791) |
| 2 | 5000 | (0.7452, 0.7462) | (0.7298, 0.7308) | (0.7521, 0.7534) | (0.9696, 0.9775) | (0.1408, 0.1424) | (0.5080, 0.5095) | (0.6216, 0.6243) | (0.4353, 0.4362) |
| 3 | 10000 | (0.7465, 0.7472) | (0.7311, 0.7318) | (0.7487, 0.7496) | (0.9668, 0.9724) | (0.1408, 0.1419) | (0.5063, 0.5074) | (0.6237, 0.6256) | (0.4519, 0.4525) |
| 4 | 30000 | (0.7464, 0.7468) | (0.7311, 0.7315) | (0.7496, 0.7501) | (0.9694, 0.9726) | (0.141, 0.1416) | (0.5072, 0.5078) | (0.6232, 0.6243) | (0.459, 0.4594) |
| 5 | 50000 | (0.7464, 0.7467) | (0.731, 0.7313) | (0.7495, 0.7499) | (0.9659, 0.9686) | (0.141, 0.1415) | (0.507, 0.5075) | (0.6236, 0.6245) | (0.4619, 0.4621) |
| 6 | 70000 | (0.7466, 0.7468) | (0.7311, 0.7314) | (0.7498, 0.7501) | (0.9658, 0.9681) | (0.1412, 0.1416) | (0.5074, 0.5078) | (0.6234, 0.6242) | (0.4585, 0.4588) |
| 7 | 100000 | (0.7463, 0.7466) | (0.7309, 0.7312) | (0.7496, 0.7499) | (0.9663, 0.9682) | (0.1411, 0.1415) | (0.5071, 0.5075) | (0.6236, 0.6243) | (0.4652, 0.4654) |
| 8 | 300000 | (0.7465, 0.7467) | (0.7312, 0.7313) | (0.7496, 0.7497) | (0.9662, 0.9673) | (0.1413, 0.1415) | (0.5073, 0.5075) | (0.6236, 0.6239) | (0.4685, 0.4686) |
| 9 | 500000 | (0.7466, 0.7467) | (0.7313, 0.7314) | (0.7497, 0.7498) | (0.9667, 0.9675) | (0.1413, 0.1414) | (0.5075, 0.5077) | (0.6236, 0.6239) | (0.4697, 0.4698) |
| 10 | 1000000 | (0.7466, 0.7466) | (0.7312, 0.7313) | (0.7497, 0.7498) | (0.967, 0.9676) | (0.1414, 0.1415) | (0.5076, 0.5077) | (0.6236, 0.6238) | (0.4729, 0.4729) |

(**c**) WDBC dataset

| Run | Sub-tables | PC | Spearman correlation | Missing values | Outliers | Class overlap | Classify accuracy | Clustering accuracy | Total quality |
| --- | --- | --- | --- | --- | --- | --- | --- | --- | --- |
| 1 | 1000 | (0.6025, 0.6058) | (0.5759, 0.5792) | (1, 1) | (0.2661, 0.2942) | (0.9243, 0.9257) | (0.4776, 0.478) | (0.5803, 0.5848) | (0.6585, 0.6651) |
| 2 | 5000 | (0.6059, 0.6076) | (0.579, 0.5807) | (1, 1) | (0.2617, 0.2738) | (0.9233, 0.924) | (0.4778, 0.4779) | (0.5826, 0.5846) | (0.7294, 0.731) |
| 3 | 10000 | (0.6035, 0.6046) | (0.5769, 0.578) | (1, 1) | (0.274, 0.2829) | (0.9229, 0.9234) | (0.4775, 0.4776) | (0.583, 0.5845) | (0.677, 0.6789) |
| 4 | 30000 | (0.6049, 0.6056) | (0.5779, 0.5785) | (1, 1) | (0.2762, 0.2814) | (0.9236, 0.9239) | (0.4778, 0.4778) | (0.5836, 0.5845) | (0.661, 0.6621) |
| 5 | 50000 | (0.6054, 0.6059) | (0.5784, 0.5789) | (1, 1) | (0.2752, 0.2791) | (0.9237, 0.9239) | (0.4777, 0.4778) | (0.5835, 0.5842) | (0.6576, 0.6584) |
| 6 | 70000 | (0.605, 0.6054) | (0.5781, 0.5785) | (1, 1) | (0.2717, 0.275) | (0.9235, 0.9237) | (0.4777, 0.4777) | (0.5838, 0.5843) | (0.6658, 0.6665) |
| 7 | 100000 | (0.6049, 0.6052) | (0.5779, 0.5783) | (1, 1) | (0.2736, 0.2764) | (0.9234, 0.9236) | (0.4777, 0.4777) | (0.5842, 0.5846) | (0.6613, 0.6618) |
| 8 | 300000 | (0.6049, 0.6051) | (0.578, 0.5782) | (1, 1) | (0.2739, 0.2755) | (0.9234, 0.9235) | (0.4777, 0.4777) | (0.584, 0.5843) | (0.6548, 0.6552) |
| 9 | 500000 | (0.6051, 0.6052) | (0.5782, 0.5784) | (1, 1) | (0.2739, 0.2751) | (0.9235, 0.9236) | (0.4777, 0.4777) | (0.5841, 0.5843) | (0.6522, 0.6525) |
| 10 | 1000000 | (0.6052, 0.6053) | (0.5784, 0.5785) | (1, 1) | (0.2737, 0.2746) | (0.9236, 0.9236) | (0.4777, 0.4777) | (0.584, 0.5842) | (0.6527, 0.6529) |

**Supplementary Table 2** Characteristics of the optimal sub-tables from each run of the DREAMER algorithm on the FHS, ADNI, and WDBC datasets. All scores are normalized within the [0,1] range, where values near 1 represent higher data quality.

(**a**) FHS dataset

| Run | Sub-tables | Rows | Columns | PC | Spearman correlation | Missing values | Outliers | Class overlap | Classify accuracy | Clustering accuracy | Total quality |
| --- | --- | --- | --- | --- | --- | --- | --- | --- | --- | --- | --- |
| 1 | 1000 | 4744 | 40 | 0.8044 | 0.7756 | 0.2964 | 1.0 | 0.8263 | 0.8801 | 0.452 | 0.6603 |
| 2 | 5000 | 4443 | 41 | 0.8259 | 0.8057 | 0.3573 | 1.0 | 0.7995 | 0.8872 | 0.4569 | 0.6917 |
| 3 | 10000 | 4946 | 42 | 0.8302 | 0.8092 | 0.3704 | 1.0 | 0.8049 | 0.8486 | 0.4469 | 0.7009 |
| 4 | 30000 | 5011 | 40 | 0.848 | 0.8327 | 0.3907 | 1.0 | 0.7577 | 0.716 | 0.3022 | 0.6981 |
| 5 | 50000 | 5112 | 41 | 0.8452 | 0.8213 | 0.3746 | 1.0 | 0.8018 | 0.8728 | 0.481 | 0.7077 |
| 6 | 70000 | 4677 | 43 | 0.8363 | 0.8191 | 0.3454 | 1.0 | 0.8114 | 0.8083 | 0.5611 | 0.7043 |
| 7 | 100000 | 4520 | 41 | 0.8422 | 0.8237 | 0.3599 | 1.0 | 0.8106 | 0.8964 | 0.3172 | 0.7078 |
| 8 | 300000 | 4855 | 40 | 0.8575 | 0.8377 | 0.3727 | 1.0 | 0.8082 | 0.8436 | 0.3162 | 0.721 |
| 9 | 500000 | 5021 | 41 | 0.8479 | 0.8311 | 0.3755 | 1.0 | 0.8038 | 0.8654 | 0.4955 | 0.7179 |
| 10 | 1000000 | 4354 | 42 | 0.8416 | 0.8225 | 0.4077 | 1.0 | 0.8048 | 0.862 | 0.4512 | 0.7232 |
| Master dataset | | **5209** | **81** | **0.7674** | **0.7363** | **0.2689** | **1.0** | **0.8071** | **0.8594** | **0.4332** | **0.6481** |

(**b**) ADNI dataset

| Run | Sub-tables | Rows | Columns | PC | Spearman correlation | Missing values | Outliers | Class overlap | Classify accuracy | Clustering accuracy | Total quality |
| --- | --- | --- | --- | --- | --- | --- | --- | --- | --- | --- | --- |
| 1 | 1000 | 2224 | 22 | 0.6859 | 0.6738 | 0.7763 | 1.0 | 0.2869 | 0.5529 | 0.6974 | 0.5274 |
| 2 | 5000 | 2211 | 22 | 0.7157 | 0.7102 | 0.7763 | 1.0 | 0.4355 | 0.6746 | 0.6525 | 0.5835 |
| 3 | 10000 | 2070 | 22 | 0.74 | 0.7379 | 0.7148 | 1.0 | 0.3995 | 0.6239 | 0.6068 | 0.5708 |
| 4 | 30000 | 2255 | 23 | 0.7161 | 0.7023 | 0.7756 | 1.0 | 0.3982 | 0.6993 | 0.4093 | 0.5781 |
| 5 | 50000 | 1989 | 27 | 0.7254 | 0.7203 | 0.7214 | 1.0 | 0.5088 | 0.7218 | 0.5203 | 0.6247 |
| 6 | 70000 | 2319 | 22 | 0.7562 | 0.7463 | 0.6903 | 1.0 | 0.4834 | 0.6724 | 0.5558 | 0.6134 |
| 7 | 100000 | 2052 | 22 | 0.6903 | 0.6874 | 0.7261 | 1.0 | 0.5892 | 0.7162 | 0.292 | 0.654 |
| 8 | 300000 | 1974 | 23 | 0.6771 | 0.6681 | 0.7362 | 1.0 | 0.5897 | 0.7148 | 0.3432 | 0.6507 |
| 9 | 500000 | 2100 | 24 | 0.6931 | 0.6962 | 0.7675 | 1.0 | 0.5419 | 0.6933 | 0.6579 | 0.6424 |
| 10 | 1000000 | 2118 | 23 | 0.6637 | 0.6616 | 0.6893 | 1.0 | 0.6506 | 0.7651 | 0.4061 | 0.6662 |
| Master dataset | | **2376** | **45** | **0.7466** | **0.7312** | **0.7497** | **0.1347** | **0.1654** | **0.5134** | **0.6012** | **0.4656** |

(**c**) WDBC dataset

| Run | Sub-tables | Rows | Columns | PC | Spearman correlation | Missing values | Outliers | Class overlap | Classify accuracy | Clustering accuracy | Total quality |
| --- | --- | --- | --- | --- | --- | --- | --- | --- | --- | --- | --- |
| 1 | 1000 | 543 | 20 | 0.6244 | 0.595 | 1 | 0.9395 | 0.9337 | 0.4723 | 0.5019 | 0.8149 |
| 2 | 5000 | 484 | 25 | 0.6427 | 0.6165 | 1 | 0.8317 | 0.936 | 0.4782 | 0.5941 | 0.8247 |
| 3 | 10000 | 569 | 17 | 0.6591 | 0.619 | 1 | 0.9875 | 0.9174 | 0.478 | 0.4645 | 0.8364 |
| 4 | 30000 | 455 | 15 | 0.6842 | 0.6607 | 1 | 0.9859 | 0.9275 | 0.4824 | 0.5918 | 0.843 |
| 5 | 50000 | 524 | 17 | 0.6755 | 0.6522 | 1 | 0.9292 | 0.9237 | 0.48 | 0.6197 | 0.819 |
| 6 | 70000 | 470 | 16 | 0.6996 | 0.6768 | 1 | 0.9243 | 0.9362 | 0.4713 | 0.4747 | 0.8355 |
| 7 | 100000 | 552 | 16 | 0.6996 | 0.6768 | 1 | 0.9245 | 0.942 | 0.4756 | 0.4719 | 0.8373 |
| 8 | 300000 | 510 | 16 | 0.6996 | 0.6768 | 1 | 0.924 | 0.9412 | 0.4784 | 0.4736 | 0.8358 |
| 9 | 500000 | 521 | 16 | 0.6996 | 0.6768 | 1 | 0.9242 | 0.9405 | 0.4798 | 0.4746 | 0.8347 |
| 10 | 1000000 | 464 | 16 | 0.6671 | 0.6527 | 1 | 0.9886 | 0.9418 | 0.4763 | 0.6201 | 0.8372 |
| Master dataset | | **569** | **30** | **0.6051** | **0.5782** | **1** | **0.0875** | **0.9315** | **0.4804** | **0.5792** | **0.6335** |
